# Unique mobile elements and scalable gene flow at the prokaryote–eukaryote boundary revealed by circularized Asgard archaea genomes

**DOI:** 10.1038/s41564-021-01039-y

**Published:** 2022-01-13

**Authors:** Fabai Wu, Daan R. Speth, Alon Philosof, Antoine Crémière, Aditi Narayanan, Roman A. Barco, Stephanie A. Connon, Jan P. Amend, Igor A. Antoshechkin, Victoria J. Orphan

**Affiliations:** 1 Division of Geological and Planetary Sciences, California Institute of Technology, Pasadena, CA USA; 2 Division of Biology and Biological Engineering, California Institute of Technology, Pasadena, CA USA; 3 Department of Earth Sciences, University of Southern California, Los Angeles, CA USA; 4 Department of Biological Sciences, University of Southern California, Los Angeles, CA USA

**Keywords:** Evolution, Ecology

## Abstract

Eukaryotic genomes are known to have garnered innovations from both archaeal and bacterial domains but the sequence of events that led to the complex gene repertoire of eukaryotes is largely unresolved. Here, through the enrichment of hydrothermal vent microorganisms, we recovered two circularized genomes of *Heimdallarchaeum* species that belong to an Asgard archaea clade phylogenetically closest to eukaryotes. These genomes reveal diverse mobile elements, including an integrative viral genome that bidirectionally replicates in a circular form and aloposons, transposons that encode the 5,000 amino acid-sized proteins *Otus* and *Ephialtes*. Heimdallaechaeal mobile elements have garnered various genes from bacteria and bacteriophages, likely playing a role in shuffling functions across domains. The number of archaea- and bacteria-related genes follow strikingly different scaling laws in Asgard archaea, exhibiting a genome size-dependent ratio and a functional division resembling the bacteria- and archaea-derived gene repertoire across eukaryotes. Bacterial gene import has thus likely been a continuous process unaltered by eukaryogenesis and scaled up through genome expansion. Our data further highlight the importance of viewing eukaryogenesis in a pan-Asgard context, which led to the proposal of a conceptual framework, that is, the Heimdall nucleation–decentralized innovation–hierarchical import model that accounts for the emergence of eukaryotic complexity.

## Abstract (web summary)

The recovery of two circularized genomes of the *Heimdallarchaeum* species from hydrothermal vent enrichment cultures reveals that these Asgard archaea carry diverse mobile genetic elements, such as an integrative viral genome and aloposons. These mobile genetic elements contain several bacteria- and phage-derived genes, modulating the shuffling of information between bacteria and archaea, and potentially influencing eukaryogenesis.

## Main

To chronicle the emergence of evolutionary innovation is a long-standing pursuit in biology. Due to scant record of reliable microscale fossils, resolving evolutionary history at the cellular scale relies primarily on molecular comparisons across present-day life, provided that phylogenetic relatives can be well delineated. Culture-independent metagenomics has substantially expanded our access to the Earth’s diverse biomes^1^, including lineages carrying genetic imprints of critical evolutionary events through deep time. The Heimdallarchaeota, previously referred to as the ancient archaea group (AAG)^2^, are one such group and the closest known relative of eukaryotes as suggested by phylogenomics^3–5^. Heimdallarchaeotes and their related lineages collectively called the Asgard archaea contain a sizeable repertoire of eukaryotic signature proteins (ESPs)^3,6,7^. However, the genetic make-up of Heimdallarchaeotes has so far only been inferred from a few metagenome-assembled genomes (MAGs), which are fragmented and suffer from uncertainty in their completeness and accuracy^3,7–12^. Mobile (genetic) elements, including transposons, viruses and plasmids, which are known to play dominant roles in evolution^13^, are frequently misassembled, omitted or misassigned during MAG assembly and binning^14^. These drawbacks propagate into uncertainties in the resolution of archaeal lineages related to eukaryotes and can obscure the drivers of evolutionary crosstalk and divergence between eukaryotes and their prokaryotic relatives.

## Results

### Circular Heimdallarchaeota genomes

**Extended Data Fig. 1:**
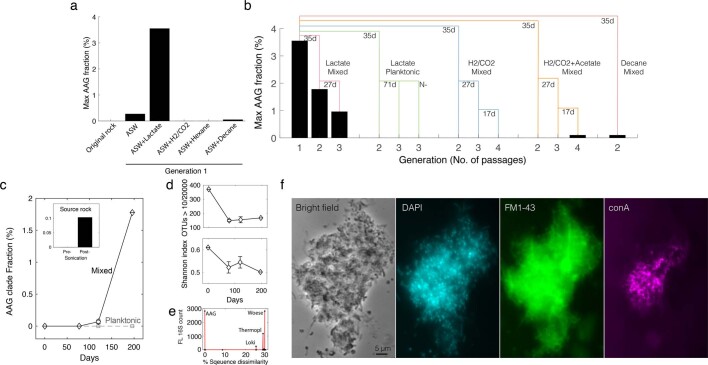
The emergence of *Ca. Heimdallarchaeum endolithica* belonging to the Ancient Archaea Group of Heimdallarchaeota in a series of incubations derived from the same rock originated from Pescadero basin. **a**. Maximum fraction of the AAG phylotype within the first-generation incubations from the rock detected within a 13-month period. **b**. Maximum fraction of the AAG phylotype within the serial dilution cultures of the initial lactate-fed culture. **c**. Amplicon sequencing of a hypervariable region in 16S rRNA gene showing the fraction of AAG phylotype in second-generation lactate-fed cultures. Mixed, mixture of rock and medium transferred from the first-generation incubation. Planktonic, only top-layer medium was transferred. **d**. Community complexity reduction over time as indicated by total operational taxonomic unit (OTU) counts (top) and the Shannon diversity index (bottom). **e**. Full-length 16S rRNA gene survey using universal archaea primers showing a single abundant AAG phylotype (*Ca. Heimdallarchaeum endolithica*) species above noise, and its 16S sequence dissimilarity (percent sequence identity difference) with other archaea in the community. Loki, a Lokiarchaeota phylotype; Thermopl, a Thermoplasmatota phylotype; Woese, a Woesearchaeia phylotype. **f**. Wide-field microscopy images of a large multispecies biofilm isolated from the lactate-fed 2^nd^-generation incubation, which was stained using DAPI (DNA), FM1-43 (membrane lipids), and concanavalin A (extracellular matrix). Imaging was repeated two times with similar observations. In c and d, error bars indicate SD. N=2, independent DNA samples extracted from the same incubation. Source data

Recovering contiguous genomes from environmental samples is notoriously challenging due to their enormous biodiversity and strain-level heterogeneity, while most known lineages have been hard to isolate due to their unresolved metabolism and/or poorly understood partner-dependent growth. We overcame these limitations by combining cultivation methods with molecular community profiling to progressively dissect environmental microbial enrichment cultures where a clonal expansion of our species of interest was accompanied by a reduction in diversity (Extended Data Fig. 1 and Methods). Using anaerobic cultivation methods, we enriched a member of the Heimdallarchaeota AAG clade from a barite-rich rock retrieved in 2017 from the Auka hydrothermal vent field (23° 57′ N, 108° 51′ W) located in the southern Pescadero Basin near the southern tip of the Gulf of California at a water depth of 3,674 m (ref. ^15^). While initially below detection, this rock-associated AAG phylotype emerged at 1–4% of the 16S ribosomal RNA gene relative abundance in 3 lactate-supplemented, anaerobic enrichment cultures incubated at 40 °C after 7 months (Extended Data Fig. 1, Supplementary Tables 1–3 and Supplementary Note 1). In an independent set of enrichments inoculated with sediments collected from the Auka site in 2018 (23° 53′ N, 108° 48′ W), alkane-supplemented anaerobic incubations at 37 °C additionally yielded a second AAG phylotype that increased in 16S rRNA gene relative abundance from 0.03 to 4–7% after 9 months (Supplementary Tables 4 and 5 and Supplementary Note 1).

**Fig. 1:**
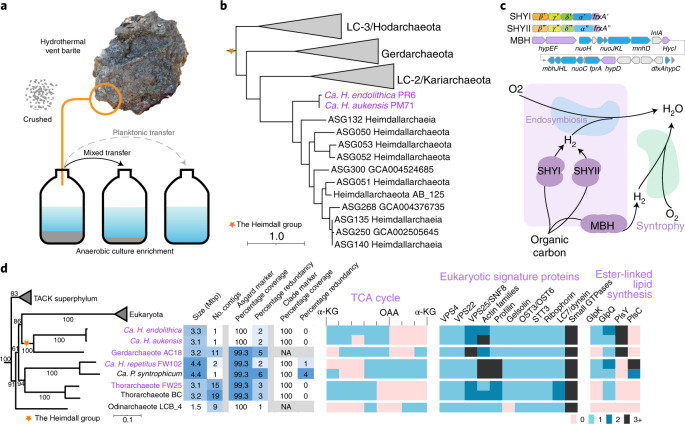
Complete genomes of *Ca. Heimdallarchaeum* spp. provide insights for eukaryogenesis. **a**, Illustration depicting the enrichment procedure of a microbial community associated with a barite-rich rock no. NA091-45R retrieved from the southern Pescadero Basin Auka hydrothermal vent field at a water depth of 3,700 m. Successive transfers of rock and media (mixed) retained the *Ca. H. endolithica* while lactate-supplemented enrichment media alone (planktonic) did not. A similar strategy was used to enrich for *Ca. H. aukensis* from the nearby sediment, substituting alkanes for lactate. **b**, Maximum-likelihood phylogeny of 57 Heimdall group Asgard archaea based on 76 concatenated archaeal marker genes. The two circular genomes of *Ca. Heimdallarchaeum* spp. are highlighted in purple. AB_125 in bold is a MAG initially described that represents the clade. **c**, A schematic illustration depicting cytoplasmic SHY and MBH operons encoded by *Ca. Heimdallarchaeum* spp. (top) and their hypothetical roles in hydrogen-based syntrophy during eukaryogenesis (bottom). For SHY operons, the four required subunits are followed by a maturation protease. For MBH operon, the electron transport genes are in blue and the maturation factors in purple. The rectangle depicts an ancient archaeon related to the *Ca. Heimdallarchaeum*; the kidney shapes depict ancient bacteria that may have formed syntrophic relations with the archaeon extracellularly or intracellularly and ultimately evolved into mitochondria. **d**, Maximum-likelihood phylogeny of Asgard archaea representatives based on a concatenation of 56 archaea–eukaryote markers from 40 genomes showing the relationship with eukaryotes followed by select genome characteristics, marker gene coverage and the presence/absence of genes encoding TCA cycle enzymes, eukaryotic signature proteins and ester-linked lipid synthesis. The genomes constructed in this study are coloured purple, with the circularized genomes indicated in bold italic. Presence/absence and gene copy number are colour-coded. α-KG, α-ketoglutarate; NA, not applicable; OAA, oxaloacetate. For **b** and **d**, A list of genomes and markers can be found in Supplementary Tables 8, 16 and 17. Source data

De novo assembly^16–18^ of Nanopore long-read and Illumina paired-end sequencing of genomic DNA recovered from these enrichments (Supplementary Table 6) resulted in complete circularized genomes of the two AAG species from the barite and sediment enrichment cultures, with genome sizes of 3.32 and 3.08 million base pairs (Mbp), respectively. The two circular AAG genomes showed 82% alignment fraction, 88% average nucleotide identity (ANI), 90% amino acid identity (AAI) and 97.9% 16S rRNA identity (Supplementary Table 7), which demarcate a clear species boundary^19^ within the same genus^20^. Thus, we propose the species names *Candidatus Heimdallarchaeum endolithica* PR6 (endo- (Greek), within; lithos (Greek), rock) and *Candidatus Heimdallarchaeum aukensis* PM71 (Auka, the local vent field) denoting their environmental origins (Fig. 1a).

### Taxonomy and metabolism

**Extended Data Fig. 2:**
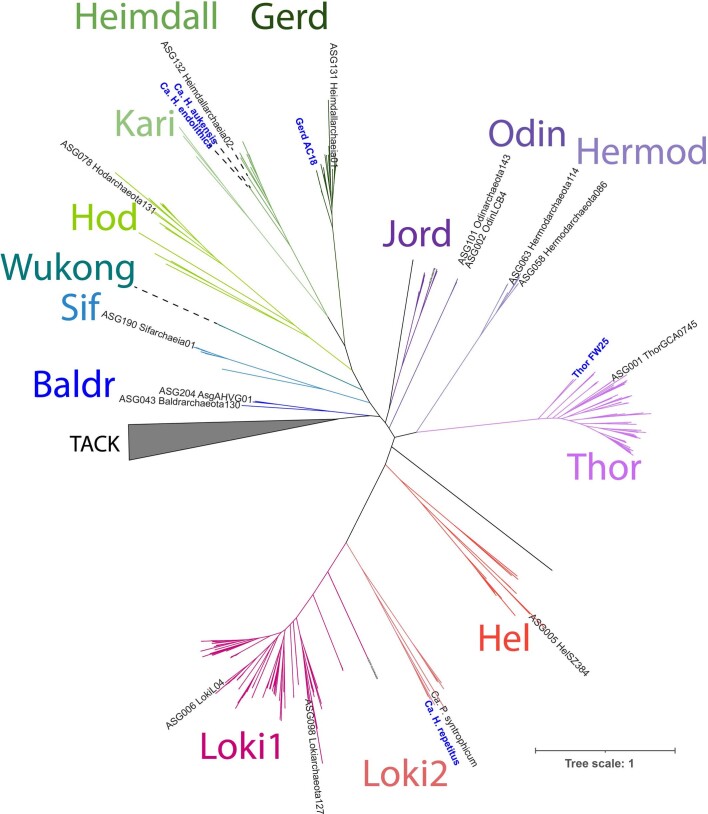
Maximum-likelihood analyses of 282 Asgard Archaea MAGs and genomes rooted using 15 TACK archaea. The different clades are labeled in different colors, with clade names indicated in the same color. MAGs selected for detailed phylogenomics analyses are annotated, with published ones in black and those constructed in this study in bold blue. Jord and Wukong clades do not yet have representatives passing the genome selection filter based on Marker coverage and genome contiguity scores. Detailed descriptions of these genomes can be found in the Supplementary Tables 8 (All Asgard archaea), S9 (Selected Asgard archaea), and S16 (TACK), Markers used can be found in Supplementary Table 17. Source data

The taxonomy of Asgard archaea is yet to reach consensus. The initial Heimdallarchaeota^3^, despite remaining monophyletic in all phylogenomic analyses, was proposed to either split into four phyla (Heimdall-, Gerd-, Kari-, Hodarchaeota)^7^ or alternatively grouped under a single order named the Heimdallarchaeia^21^. In this study, we collectively refer to them as ‘the Heimdall group’. Phylogenomic analyses based on 76 concatenated ribosomal proteins show that the *Heimdallarchaeum* spp. constitute a deeper-branching clade related to the previously described MAG AB_125 (ref. ^3^), well placed under ‘*Heimdall*’ in all proposed classification strategies (Fig. 1b and Extended Data Fig. 2). Additionally, we also identified a fragmented MAG B53_G16^22^ (299 contigs, 1.67 Mbp, approximately 50% complete) from the Guaymas Basin, formerly assigned under the Pacearchaeota, which we now designate as a strain of *Ca. H. endolithica*, with an average ANI of 97.5% compared with our PR6 strain.

**Extended Data Fig. 3:**
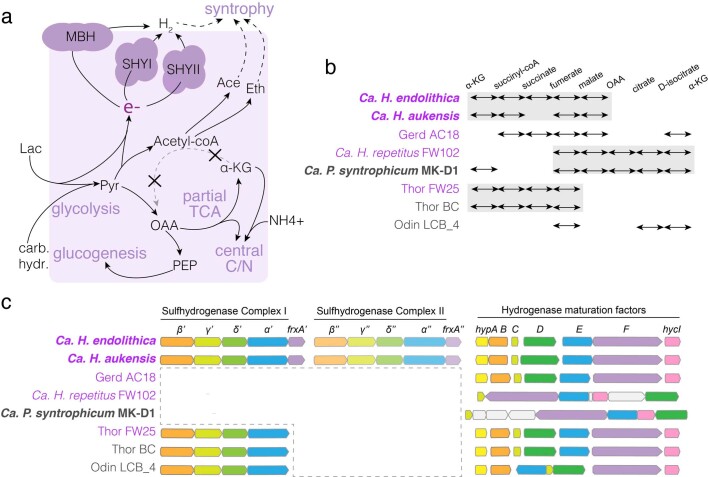
Genome-based metabolic predictions of *Ca. Heimdallarchaeum spp*. and comparisons with other contiguous, near-complete Asgard Archaea MAGs. **a**. Illustration of metabolic reconstruction highlighting hydrogen metabolism and tricarboxylic acid (TCA) cycle. Abbreviations: α-KG, α-ketoglutarate; OAA, oxaloacetate; SHY, sulfhydrogenase (cytosolic hydrogenase); MBH, membrane-bound hydrogenase; lac, lactate; carb.hydr., carbohydrate; Pyr, pyruvate; PEP, phosphoenolpyruvate; Ace, acetate; Eth, ethanol. **b** and **c**. Enzymes involved in TCA cycle reactions (b) and cytosolic hydrogen evolution (c) in each genome/MAG representatives of Asgard archaea.

*Ca. Heimdallarchaeum* spp. are predicted to garner energy by anaerobically oxidizing organic substrates via processes involving a partial tricarboxylic acid (TCA) cycle and, given the absence of discernible terminal electron accepting pathways, dissipating electrons via H_2_ production (Extended Data Fig. 3a). They each encode one membrane-bound hydrogenase (MBH) complex and two cytosolic sulfhydrogenase complexes (SHYI and SHYII) (Fig. 1c). Hydrogen has been hypothesized to act as a syntrophic intermediate bridging archaea and bacteria before the engulfment of mitochondrial ancestor by an (Asgard) archaeal ancestor of eukaryotes^4,23–25^. Indeed, in the recent description of *Ca. Prometheoarchaeum syntrophicum*, MBH associated with unusual membrane extensions were hypothesized to facilitate cell–cell contact and hydrogen exchange with syntrophic partner bacteria^23^. Following from this concept, we postulate that cytosolic hydrogen generation by SHY, as found in the *Ca. Heimdallarchaeum* spp., could impose a selective advantage for a hydrogen-dependent endosymbiotic strategy (Fig. 1c).

### Eukaryotic signatures

**Extended Data Fig. 4:**
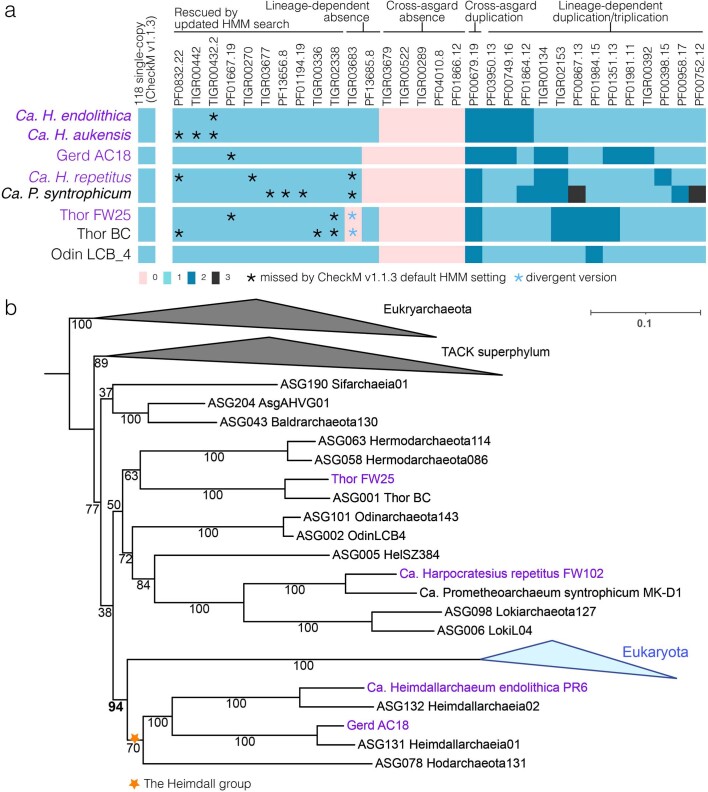
Marker determination and phylogeny of expanded representatives of Asgard archaea. **a**. Differential distributions of putatively single-copy archaea marker genes in initially selected genomes/MAGs, which show cross-asgard and clade-specific marker coverage features. **b**. Maximum-likelihood phylogeny of an expanded selection of asgard archaea MAGs in relation to Euryarchaeota, TACK, and Eukaryota. *Ca. H. aukensis* was omitted to improve evenness in the taxonomic selection here due to its close relation with *Ca. H. endolithica*. Detailed descriptions of the 51 genomes used in the analyses can be found in Supplementary Tables 9 (Selected Asgard archaea) and S16 (TACK+Eukaryotes). Markers used can be found in Supplementary Table 17. Purple indicates genomes and MAGs constructed in this study. Source data

One of the many challenges of resolving the relationship between archaea and eukaryotes is the curation of representative, high-quality genomes across lineages at their interface. To this end, we verified the complete marker gene coverage of the *Ca. Heimdallarchaeum* spp. as well as six other highly contiguous Asgard archaea genomes (Extended Data Fig. 4a, Methods and Supplementary Note 2). They include three previously described^3,23,26^ and three assembled in this study from our enrichment cultures—a Lokiarchaeote that we have named *Ca. Harpocratesius repetitus* FW102, a Thorarchaeote FW25 and a Heimdall group Gerdarchaeote AC18 (Fig. 1d). Notably, the dual-contig assembly *Ca. H. repetitus* FW102, which relates to *Ca. P. syntrophicum* MK_D1 at the family level, contains two complete sets of 16S/23S rRNA genes, potentially relevant to their growth strategies in the environment^27^.

These complete genomes confirmed that many of the previously described ESPs^3,6^ are distributed universally across known Asgard phyla (Fig. 1d), specifically genes involved in (1) membrane remodelling (endosomal sorting complexes required for transport components VPS4/VPS22/VPS25), (2) cytoskeleton organization (actin, profilin and gelsolin (except in Odin LCB_4)), (3) protein *N*-linked glycosylation (OST3/STT3/ribophorin) and (4) intracellular trafficking (roadblock/LC7/dynein family and a large repertoire of small GTPases). On the other hand, enzymes involved in the synthesis of ester-linked phospholipids, which are critical for closing the ‘lipid divide’ between the Archaea and Eukaryota domains^23,26^, show a mosaic distribution across the Asgard archaea lineages (Fig. 1d). For example, both *Ca. Heimdallarchaeum* spp. in our study lack 1-acyl-sn-glycerol-3-phosphate acetyltransferase involved in the attachment of the second fatty acid chain to the glycerol backbone^28^.

Maximum-likelihood analysis using a previously described approach based on the SR4 model^3,29^ and a concatenation of a complete set of 56 single-copy markers, indicates a close relationship between the Heimdall group archaea, which include the *Heimdallarchaeum* spp. and eukaryotes (Fig. 1d). This supports a parsimonious topology, reported in multiple studies^3,5,7^. We additionally produced a set of customized Asgard-specific Hidden Markov Models (HMMs) (Supplementary Data 1) that complement existing Archaea-specific HMMs along with a set of filtering parameters (Methods and Supplementary Tables 8 and 9) as resources. Maximum-likelihood analyses of a greater diversity of Asgard archaea^7,11,12,16^ that were selected through the framework described above (19 of 282 evaluated MAGs shown in Extended Data Fig. 2) further verified the phylogenetic topology, placing the Heimdall group closest to eukaryotes (Extended Data Fig. 4b). We note that statistical model selection, taxonomic evenness and assumptions with rooting represent ongoing debates for deep phylogeny^5,7^. The circularized genomes and resources described in this study may assist with future analyses of the Asgard archaea using a broader range of statistical parameters and emerging high-quality genomes.

### Abundant repetitive features

**Fig. 2:**
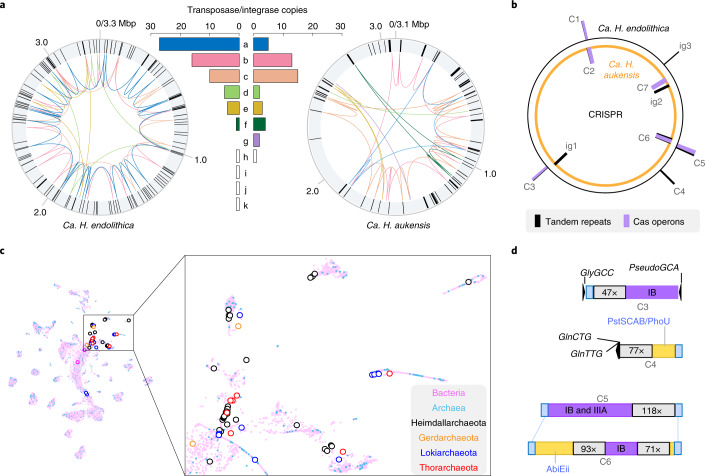
Circular *Heimdallarchaeum* genomes reveal abundant repeats belonging to complex networks of transposases/integrases and CRISPR–Cas operons. **a**, Representation of the circularized genomes of *Ca.* *H. endolithica* and *Ca.* *H. aukensis* where the black bars in the outer rings denote non-tandem repeat sequences identified using a cut-off of 100 bp alignment length and 95% sequence identity. Inner networks connect the transposases/integrases belonging to the same family, with the copy numbers of each family (a–k) shown in the bar chart using the same colour scheme. **b**, Schematic showing the genomic distribution of CRISPR–Cas operons (C1–C7) and intragenic tandem repeats (ig1–3) across the two circular genomes of *Heimdallarchaeum* spp. **c**, Alignment score matrix clustering of diverse transposases/integrases showing their evolutionary exchange across archaeal and bacterial domains. Each marker represents a sequence that has been colour-coded by its taxonomic affiliation with the Bacteria domain in pink and the Archaea domain in blue. Highlighted in the open circles are the identified transposases/integrases associated with Heimdallarchaeota, Gerdarchaeota, Lokiarchaeota and Thorarchaeota. **d**, The specific operon structures of CRISPR–Cas and their mobile element signatures, including integration at tRNA genes (C3 and C4) and complete local displacement (C5 and C6), are shown to the right. The text in the purple boxes indicates the Cas operon types; the numbers in the grey boxes denote the number of repeats. Yellow indicates neighbouring unconserved genes; blue indicates flanking sequences conserved between two *Ca. Heimdallarchaeum* genomes.

Our approach retained a substantial number of non-tandem repeats (3% of genome lengths) and tandem CRISPR or intragenic repeats (212 and 262 counts) within the circular *Ca. Heimdallarchaeum* spp. genomes (Fig. 2a,b). This is notably more prominent relative to the recently constructed circular genomes of *Ca. P. syntrophicum*^23^, where no tandem repeats and only 1% of non-tandem repeats were observed.

Non-tandem repeats in the *Ca. Heimdallarchaeum* spp. overlap prominently with one of the most pervasive mechanisms of gene transfer within and between genomes, that is, a total of 11 families of transposases/integrases, 7 of which have multiplied and transposed to result in up to 27 copies within an individual genome (Fig. 2a). These and other transposases/integrases found in Asgard archaea primarily cluster with various small families within the 96,367 transposase/integrase sequences recovered from the prokaryotic Genome Taxonomic Database (GTDB)^30^ (Fig. 2c). Despite the under-representation of archaeal sequences in public databases and in the transposase/integrase dataset in this study, they have representatives in almost all clusters. The intermingled evolutionary relationship between archaeal and bacterial transposases/integrases documented in this study can potentially be both the result of, and contributor to, the gene flow observed between these two domains^31–33^.

**Fig. 3:**
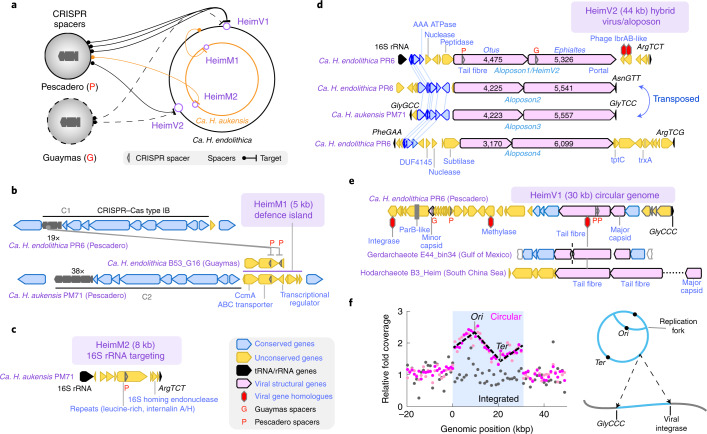
Unique Heimdallarchaeal mobile elements with viral and transposable features. **a**, CRISPR-targeted mobile elements in the two *Heimdallarchaeum* genomes with viral features (HeimV1 and HeimV2) and without viral features (HeimM1 and HeimM2). The orange/black solid/dashed lines highlight connections between the CRISPR spacers recovered from two geographically distant vent sites in the Gulf of California (Pescadero and Guaymas Basins) to their matching target (protospacers) within the two genomes derived from Pescadero. **b**–**e**, Gene synteny of HeimM1 and HeimM2, with legend as shown in **c**. All tRNA genes were annotated with amino acid abbreviations followed by their anticodon, for example, GlyCCC. **b**, HeimM1 was integrated next to C2 Cas gene operon and is targeted by a pair of C1 CRISPR spacers. **c**, HeimM2 contains a repeat peptide-encoding gene that was targeted by a Pescadero Basin spacer. **d**, HeimV2 was compared with its related transposons discovered in this study—aloposons. **e**, HeimV1 was compared with two other MAGs belonging to different clades within the Heimdall group. **f**, Left, normalized sequencing coverage around HeimV1, highlighted in the blue background. Light pink and dark pink show single- and paired-end sequencing on the same DNA sample; grey shows the paired-end sequencing data of a second DNA sample from a different culture. The dashed line highlights the V shape, a signature of the bidirectionally self-replicating circular virus genome. Each dot is an average value binned at a 1 kb interval. Right, illustration depicting the integrated (bottom) and replicating (top) circular states indicated by the plot on the left. The arrows indicate the genomic integration next to the tRNA gene GlyCCC by a viral integrase.

**Extended Data Fig. 5:**
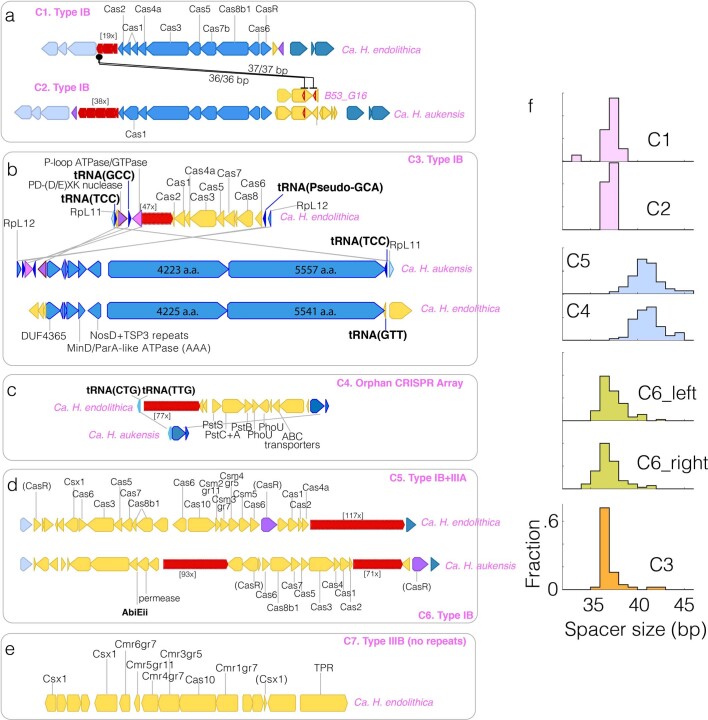
CRISPR/Cas systems in *Ca. Heimdallarchaeum spp*. **a-e**. Schematic showing the gene synteny of the CRISPR/Cas systems (serial numbers and operon types are in bold pink) and their alignments between the two genomes. Genes conserved between the two genomes are labeled in various shades of blue and purple to assist visualization. Genes only appearing in one of the genomes are in yellow. Red indicates CRISPR arrays. Array sizes are indicated by the number of repeats such as [77x]. In **b**, The *Aloposons* with giant genes are also shown to illustrate their site-specific integration. **f**. Size distribution of spacers in each CRISPR array. Source data

The circular genomes of *Ca. Heimdallarchaeum* spp. contain seven CRISPR–Cas systems (Fig. 2b), including five complete operons (labelled C1–3, 5, 6), one array-free operon (C7) and one orphan array (C4) (see Extended Data Fig. 5 for the complete gene organizations). Contrasting the overall gene conservation between the two genomes, these CRISPR–Cas systems exhibit strong variability and site-specific integration (Fig. 2d). For example, C5 and C6 exhibited a complete local operon swap, while C3 and C4 were integrated immediately next to transfer RNA genes, a feature often exploited by bacteriophages^34^ and other Heimdallarchaeal mobile elements (see examples in Fig. 3 below).

### CRISPR–Cas-guided discovery of mobile elements

We recruited a total of 1,565 Heimdall-associated CRISPR spacers in our Pescadero metagenomes constructed in this study and previously published Guaymas metagenomes (Methods). They revealed eight protospacers within four distinct mobile elements, which are hosted by *Ca. Heimdallarchaeum* spp. and are unrelated to any previously reported mobile elements (outlined in Fig. 3a). We named them Heimdallarchaeal mobile elements HeimM1 and HeimM2 and Heimdallarchaeal viruses HeimV1 and HeimV2, respectively.

HeimM1, detected within the sediment-hosted *Ca. H. aukensis*, is a C2-associated small defence island encoding an efflux pump *CcmA* and contains a protospacer that matches a spacer at the same genomic locus in the rock-hosted *Ca. H. endolithica* PR6 C1 (Fig. 3b). Such a territorial dispute within the genome, as well as the site-specific integrations of CRISPR–Cas outlined above, exemplify the emerging view that defence systems are mobile elements themselves^35^ and contribute to gene flow between habitats.

HeimM2 (8 kbp) encodes an internalin-like, leucine-rich repeat peptide and an enzyme homologous to rRNA self-splicing homing endonucleases (Fig. 3c). The latter are typically found as group I introns embedded within rRNA genes and are considered selfish elements. In this study, this gene was part of a mobile element inserted exactly between the only copy of the 16S rRNA gene and the tRNA gene *ArgTCT*, suggesting that it has likely been co-opted by HeimM2 for site-specific integration at this site.

**Extended Data Fig. 6:**
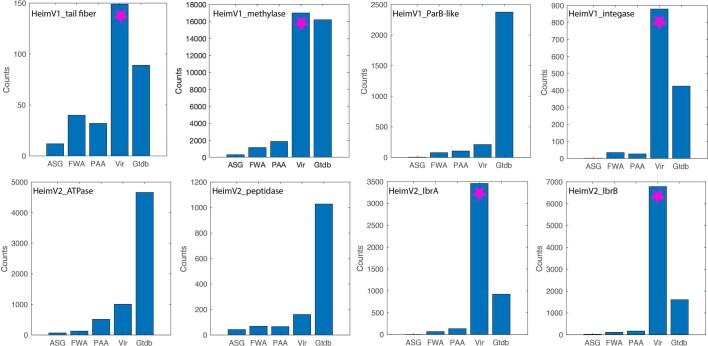
Numbers of sequences homologous to some of the proteins encoded by Heimdallarchaeal viruses HeimV1 and HeimV2. Magenta stars indicate enrichments in viral database. The homology search was carried out using diamond v2.0.6 using a e-value cutoff of 10^-3^. ASG, asgard archaea genomes; FWA, in-house metagenomic assemblies of microbial communities in Pescadero basin incubations; PAA, publicly available and published metagenomic assemblies of microbial communities in Guaymas basin sediment; Vir, IMGVR3 viral database; Gtdb, genomic sequences from GTDB v202. See methods and supplementary tables for the details of these datasets. Source data

The putative integrated viruses HeimV1 and HeimV2 are both found in *Ca. H. endolithica*. Each encodes proteins with homologues preferentially found in the viral database IMG/VR v.3^36^ compared to the microbial genome database GTDB v.202, and viral structural proteins predicted by machine learning-based annotations (PhANNs^37^) (Fig. 3d,e and Extended Data Fig. 6).

**Extended Data Fig. 7:**
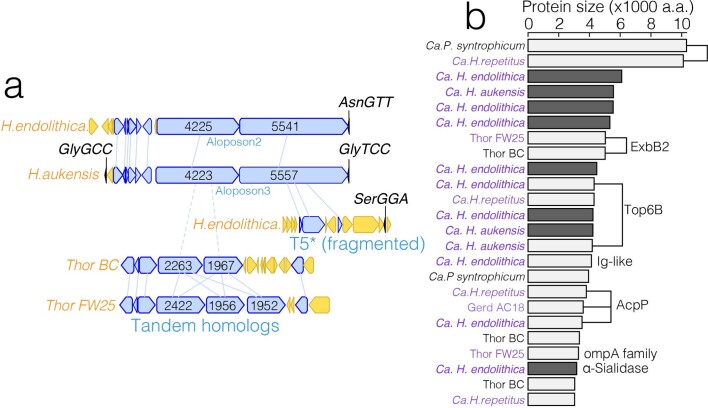
Giant proteins encoded by Asgard archaea. a. Gene synteny showing 1) an additional genomic region with truncated, fragmented sequences homologous to one of the giant genes in Aloposons, and 2) tandem giant genes which show high homologies with their neighbors are found in Thorarchaeotes, and are distantly related to one of the two giant genes in Aloposons. b. Giant proteins larger than 3000 a.a. encoded by selected Asgard archaea representatives. In dark grey are part of the Aloposons. Functional domains as identified through conserved domain database (CDD) analyses are indicated on the right. Purple indicates genomes constructed in this study.

HeimV2 (44 kbp), integrated at the same site as HeimM2, may be a hybrid between a virus and a previously undescribed class of transposons, which we tentatively call *aloposons*, in reference to the twin giants Aloadae in Greek mythology. They share the following features (Fig. 3d). First, they all contain tandem genes encoding proteins 3,000–6,000 amino acids in size, which we refer to as *Otus* and *Ephialtes*, the Aloadae twins. Second, they all integrate at different tRNA sites downstream of the giant genes. *Aloposon2* in *Ca. H. endolithica* and *Aloposon3* in *Ca. H. aukensis* represent a highly conserved element that has transposed from one tRNA site to the other during its coevolution with its host. Third, they all encode four consecutive genes upstream of the giant genes, including a gene encoding a bacterial MinD/ParA-like AAA family ATPase. Additionally, we found tandem giant genes in two Thorarchaeota MAGs showing distant homology to the *Heimdallarchaeum* giant proteins, as well as many unrelated giant genes across the Asgard archaea, some of which may also be part of Asgard mobile elements (Extended Data Fig. 7).

Putative virus HeimV1 (30 kbp) is a circular element with a highly polycistronic gene arrangement and an enrichment in nucleic acid-processing enzymes, viral structural proteins and viral gene homologues (Fig. 3e). As shown in Fig. 3f, HeimV1 exists in two states. Besides the genome-integrated lysogenic state found in one of the incubations, where its sequencing read abundance was at the same level as its genomic neighbourhood, in another enrichment incubation, HeimV1 showed an anomalously high read abundance relative to the host *Ca. H. endolithica*, suggestive of active replication. PCR and Sanger sequencing further confirmed the circularized state of HeimV1 as well as its integration between the host transposase and tRNA genes. Furthermore, the detailed sequencing read abundance profile across HeimV1 shows the characteristic V shape of an unsynchronized, bidirectionally self-replicating population of circular DNA elements (Fig. 3f). Such a well-defined profile can only emerge if the replications in each HeimV1 circular element initiate at a defined origin of replication.

The mobile elements described above also influence ecosystems beyond the southern Pescadero Basin vent system. CRISPR spacers targeting HeimV1 and HeimV2 were detected in metagenomes from the Guaymas Basin^22^, a hydrothermal vent site 400 km northwest of the southern Pescadero Basin. The Pescadero-derived mobile element HeimM1 in *Ca. H. aukaensis* also exists in the *Ca. H. endolithica* B53_G16 MAG assembled from the Guaymas Basin. Furthermore, HeimV1-related proviruses encoding tail fibre protein homologues are also found in the Heimdall group MAGs from the Gulf of Mexico in the Atlantic (Gerdarchaeota clade E44_bin34 (ref. ^9^)) and from the South China Sea (Hodarchaeota clade B3_Heim^10^) on the other side of the Pacific (Fig. 3e). Notably, the contig in the E44_bin34 MAG maintains the same gene synteny around the tail fibre gene as in HeimV1, albeit with only approximately 30% sequence homology. These observations indicate the expansive distribution of these mobile elements in diverse lineages of Heimdall group archaea across a large geographical range in deep sea ecosystems.

### Diverse evolutionary origins of Heimdallarchaeal viruses

**Fig. 4:**
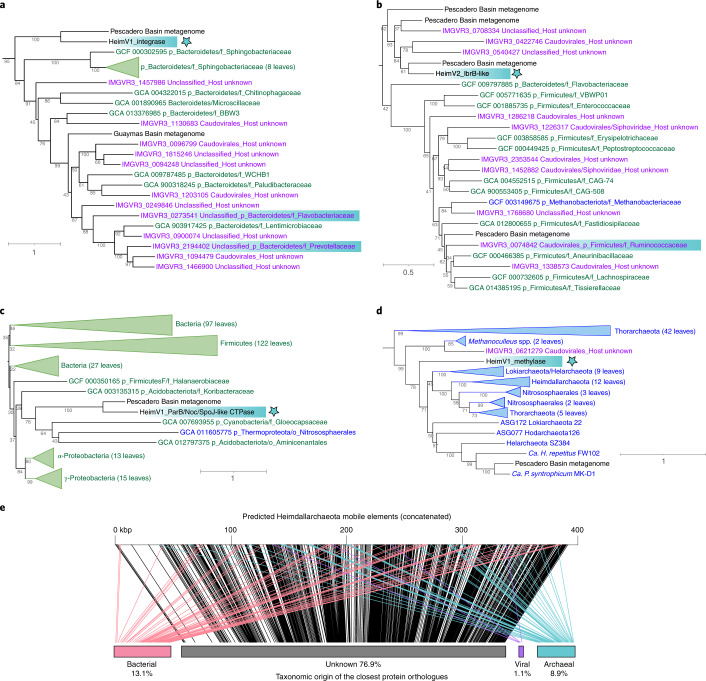
Gene phylogeny of Heimdallarchaeal viruses and other mobile elements. **a**–**d**, Maximum-likelihood analyses showing the evolutionary relationship between the proteins encoded by the viral-like mobile elements HeimV1 and HeimV2 (bold black, marked by a blue star) with known viruses (magenta), bacteria (green), archaea (blue) and sequences from the Pescadero and Guaymas Basins metagenome assemblies (black). Highlighted with blue backgrounds are viruses with identified hosts. Bootstrap values are listed. The numbers of proteins selected for the phylogenetic analyses are 172 (**a**), 142 (**b**), 285 (**c**) and 87 (**d**). The serial numbers of the microbial and viral genomes are indicated in the figures and source data files. **e**, Schematic representation of the 56 contigs from the mobile elements targeting Heimdallarchaea mapped to the closest known homologues in bacteria (pink), archaea (teal) or viruses (purple) through protein orthologue analyses using eggNOG v.5.0. MGE contigs are ranked by size from large (44 kbp) to small (2.8 kbp) and concatenated. The percentages of each taxonomic group measured in the total gene lengths are indicated. Source data

**Extended Data Fig. 8:**
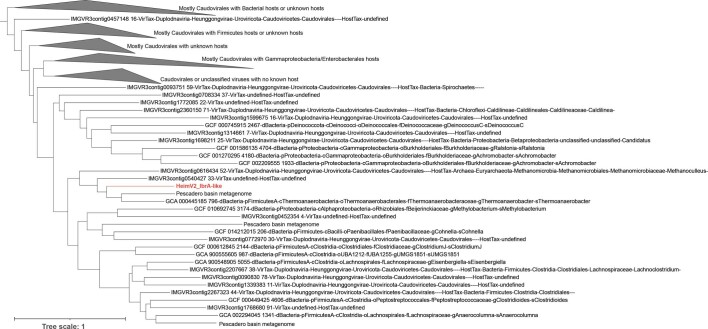
Maximum-likelihood analyses of HeimV2 IbrA-like protein. The branch names are as follows: For viruses, serial numbers followed by viral taxonomy then followed by host taxonomy if available. For microbial genomes, serial numbers followed by taxonomy. In total, 147 proteins were included in the analyses. Source data

Phylogenetic analyses of viral genes indicate that HeimV1 and HeimV2 share their evolutionary origins with bacteriophages. As shown in Fig. 4a, the viral integrase of HeimV1 is phylogenetically most closely related to integrases found in environmental bacteriophages identified to be hosted by the phylum Bacteroidetes, along with integrases found in seven families of Bacteroidetes and other viruses with microbial hosts that are unidentified. Similarly, independent phylogenetic analyses of homologues of proteins affiliated with prophage transcriptional regulators, IbrA and IbrB, which are encoded by HeimV2 simultaneously found their closest relatives in bacteriophages or unidentified elements targeting diverse members of phylum Firmicutes (Fig. 4b and Extended Data Fig. 8).

While most viruses encoding genes related to HeimV1 and HeimV2 are unclassified, several belong to the order Caudovirales, including members of the family Siphoviridae. Well-studied members of Caudovirales are known to be tailed bacteriophages packaging double-stranded DNA, in line with the machine learning-based predictions of tail fibres in both HeimV1 and HeimV2 (>90% confidence; Fig. 3d,e).

Heimdallarchaeal viruses and other mobile elements associated with the Heimdall group archaea are predicted to have origins in both bacteria and archaea. For example, HeimV1 encodes a protein with two unknown domains flanking a full-length CTPase homologous to Noc/ParB/SpoJ-like proteins that bind DNA and regulate bacterial cell division (Fig. 4c). On the other hand, the HeimV1 methylase gene appears to have evolved from the Asgard archaea and is potentially involved in evading host detection (Fig. 4d). Phylogenetic analysis suggests that divergence of this viral methylase from its host was an ancient event that occurred before the divergence between the Heimdall and Loki group archaea, estimated to have taken place around two billion years ago^38^.

A survey of *Heimdallarchaeum*-associated protospacers within the entire Pescadero/Guaymas metagenomic dataset yielded 56 total contigs belonging to the putative Heimdall group mobile elements (Supplementary Data 2). Most coding sequences (76.9%) have no apparent homology with known microorganisms and viruses, while another 13.1% have homologues in diverse bacteria (Fig. 4e), which is higher than the 8.9% archaeal fraction. This further suggests that mobile elements and viruses may play a prominent role in shaping the evolution of Heimdallarchaeota by introducing functional innovations of bacterial origin.

### Asgard–eukaryote parallelism in bacterial gene import

**Fig. 5:**
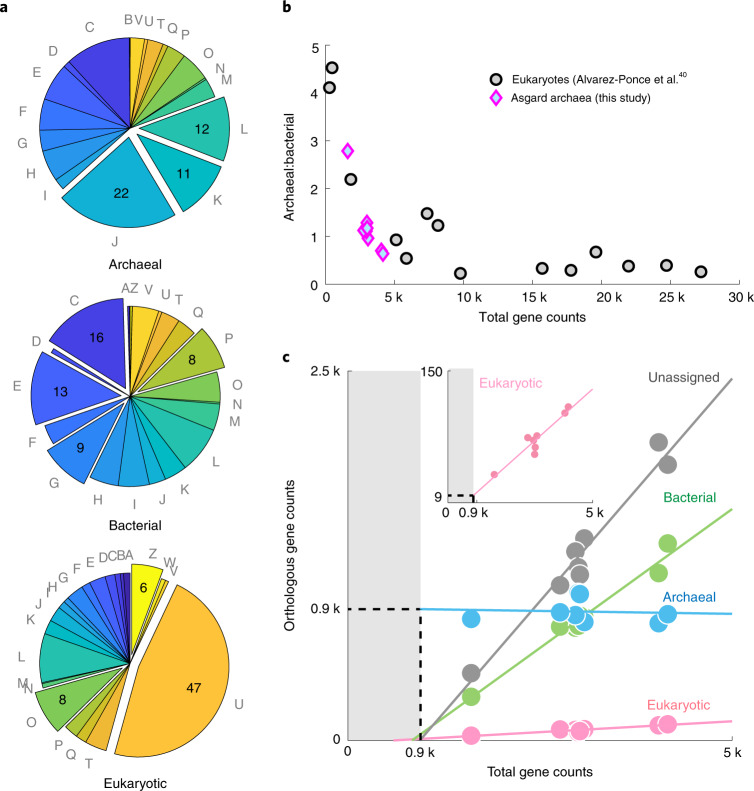
Functional and taxonomic profiling of gene content cross Asgard archaea. **a**, COG classification of genes within the Asgard archaea subdivided into closest taxonomic groups using eggNOG. The expanded wedges in each pie chart highlight the top categories preferentially enriched in the taxonomic group than other groups. They respectively indicate translation (J), transcription (K), replication and repair (L), energy production and conversion (C), the metabolism and transport of amino acids (E), carbohydrates (G) and inorganic ions (P), intracellular trafficking and secretion (U) and cytoskeleton (Z) and protein modification (O). The remaining groups can be found in Tatusov et al.^40^. The numbers indicate the percentages. Note that proteins with unknown function are excluded from each pie chart. **b**, The archaeal:bacterial gene ratio decreases with increasing genome size in both Asgard archaea (this study) and eukaryotes (data from Alvarez-Ponce et al.^41^). **c**, Numbers of genes related to different taxonomic groups in relation to the total number of genes in the representative genomes of the Asgard archaea indicate different scaling properties. The solid lines represent the linear fit of the data. The dashed lines represent the extrapolated base number of archaeal genes. Unassigned means that no homology was found in the genome database. Inset, Expanded view of the genes encoding ERPs. Source data

To understand the consequence of cross-domain gene flow in the evolution of Asgard archaea, we performed protein orthology-based functional and taxonomic profiling^39^ of the proteomes encoded by the complete genomes in this study. Functional analyses of the Asgard archaeal proteome based on clusters of orthologous groups (COGs)^39,40^ revealed distinct categories of genes that are associated with different taxonomic groups (Fig. 5a). The Archaea-related proteins in Asgard archaea were predominantly represented by information processing functions, including translation (J), transcription (K) and replication and repair (L), which is similar to the key archaeal modules inherited by eukaryotes^41^. By contrast, the annotated bacteria-related proteins were preferentially enriched in metabolic functions, including energy production and conversion (C) and the metabolism and transport of amino acids (E), carbohydrates (G) and inorganic ions (P). Different from both the above groups, nearly half of eukaryote-related proteins within the Asgard genomes were dedicated to intracellular trafficking and secretion (U), and cytoskeleton (Z) and protein modification (O) functions.

The import of bacterial genes into archaea and eukaryotes have been independently explored^31,32,41,42^. In this study, we show that the inheritance of information processing from the Archaea and metabolic functions from the Bacteria domain in the Asgard archaea is very similar to the signature of the eukaryotic genome profile. Strikingly, the archaeal:bacterial gene ratio forms an inverse relation with the genome size in Asgard archaea that is quantitatively comparable with previous characterizations across eukaryotes^41^ (Fig. 5b). Such a quantitative agreement on their genome size dependence suggests that the bacterial import of genomic material into eukaryotes may not necessitate an independent mechanism (such as endosymbiosis^42^) or a dramatically different selective force from their closest archaeal relatives. Instead, genome size control alone may be sufficient to account for the over-representation of bacterial genes in some eukaryotes^43^.

### Domain-specific scaling of gene flow

**Extended Data Fig. 9:**
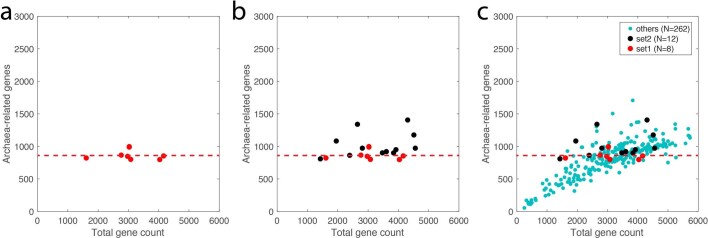
Scaling property of gene flow is obscured by fragmented genomes of varying quality. The plots show the number of Archaea-related genes in relation to the total gene counts in the Asgard archaea genomes. **a**. only the 8 genomes investigated in detail in this study. All genomes have less than 20 contigs and with verified coverage of all archaeal markers. **b**. In addition to a, an additional 12 genomes were added (in black), which contain no more than 100 contigs with a loosened completeness scores as shown in Supplementary Table 9. Since marker redundancy differs among lineages, contamination level is hard to assess. **c**. In addition to b, all other 262 published Asgard archaea genomes were added (in green). This indicates a severe deviation from the invariable relation shown in a, but instead show a near linear relation. This can be understood that in either incomplete or contaminated genomes, all types of genes have equal possibility to be retained. For example, the 1.5Mb Odinarchaeote genome contains the similar number of Archaea-related genes (~900) as a Lokiarchaeote genome sized 4.4Mb. However, if a Lokiarchaeote is fragmented into 300 contigs and only 1.5Mb in total length is randomly binned into a MAG, the latter will roughly contain ~ 300 Archaea-related genes. Hence, the type of relation shown in (a) can only be captured in highly confident, complete genomes. Legend for all panels is shown in c. Source data

Different scaling laws appear to govern the fluidity of genes with different taxonomic origins within the Asgard archaea. The total number of genes with closest orthologues in Archaea were remarkably invariable at approximately 900 genes across all Asgard archaeal representatives that span a threefold difference in genome size, from 1.5 Mbp in Odin LCB_4 to 4.4 Mbp in Lokiarchaeotes (Fig. 5c). While the archaeal reference database is currently significantly smaller than the bacterial one, which likely caused an underestimation of the exact number of archaea-related genes, the trend cannot be explained by such a database bias. One the other hand, we found that genome completeness and accuracy is key to capturing this feature since it is otherwise entirely obscured in Asgard genomes of variable completeness and contamination levels (Extended Data Fig. 9). By contrast, the bacterial, eukaryotic and taxonomically unassigned fractions of the genome increased linearly with the remaining portion of the genome. These scaling properties suggest a fundamental difference in the evolutionary plasticity between conserved archaeal ‘core’ genes and other fractions of the gene content with different evolutionary origins among the Asgard archaea.

### Decentralized eukaryotic innovation

Eukaryote-related proteins (ERPs) capture present-day Asgard–Eukaryota protein orthologues that are estimated to be most closely related to each other. They include, but are not restricted to, previously investigated ESPs^3,6,7^—loosely defined as eukaryotic proteins with no archaeal or bacterial homologues in the predicted last eukaryotic common ancestor (LECA)^44^. Our analyses show that the scaling property of ERPs is similar to bacteria-related but not archaea-related proteins (Fig. 5c), prompting us to explore their evolutionary fluidity across Asgard archaea lineages.

**Fig. 6:**
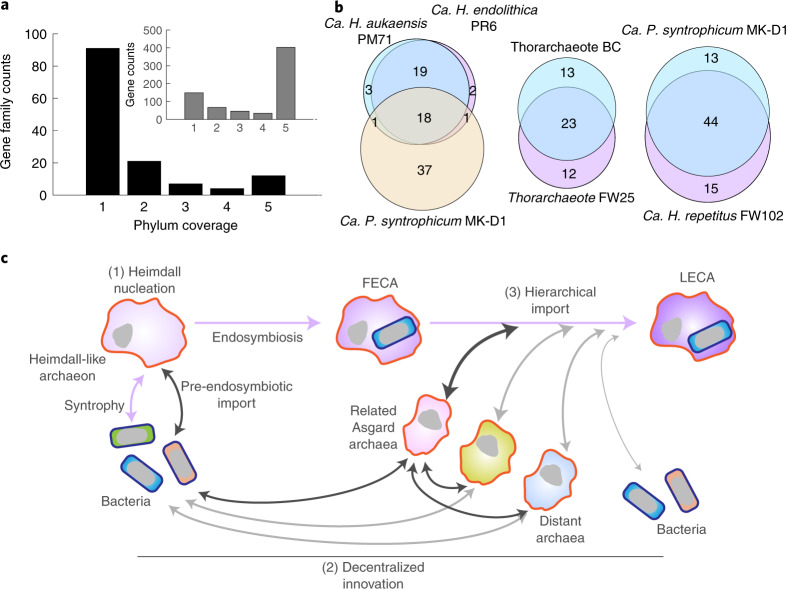
Distribution of ERP genes and the hypothesized HDH model for eukaryotic origin. **a**, Presence of various ERP gene families across the selected representatives as shown in Fig. 1d, which belong to five candidate Asgard archaeal phyla—Heimdallarchaeota, Gerdarchaeota, Lokiarchaeota, Thorarchaeota and Odinarchaeota. Inset, Total gene numbers belonging to the gene families shown in **a**. **b**, Venn diagrams showing the ERP gene families shared between lineages of different phylogenetic distances, including three circular genomes (left), two Thorarchaeota members related at the family level (middle) and two members of the Lokiarchaeota related at the family level (right). **c**, The proposed HDH model provides a conceptual framework for the process of genome acquisition during early eukaryotic evolution. Key steps include a Heimdall-like ancestral archaeon with a simple genome engaged in endosymbiosis with a bacterium to establish the FECA. FECA then acquired innovations across the tree of life via an extensive gene import, most frequently, and often indirectly, through close closely related Asgard archaea, to ultimately orchestrate the LECA. The pink arrows indicate several major phases during early eukaryotic evolution. The dark arrows indicate horizontal transfer events from or via Asgard archaea into the eukaryotic genomes. The grey arrows indicate other horizontal transfer events that occurred and contributed to the eukaryotic genomes, although to a lesser extent. Source data

Beyond the ESPs described above, which are shared by all Asgard archaea (Fig. 1d), we found diverse families of ERPs existing in only one or two of the Asgard clades examined in this study (Fig. 6a). Comparison of the circular genomes of *Ca. Heimdallarchaeum* spp. and the Lokiarchaeote *Ca. P. syntrophicum* revealed fewer than half of their ERP families being shared, notably with members of the *Heimdallarchaeum* harbouring fewer ERPs overall, despite their closer phylogenetic relationship with eukaryotes (Fig. 6b). Furthermore, even species related at the genus (*Ca. Heimdallarchaeum* spp.) or family levels (within Thorarchaeota/Lokiarchaeota) have apparent differences in their ERP pools (Fig. 6b). Such a high mobility of ERPs in the recent evolutionary history of Asgard archaea suggests that many of these genes are involved in the auxiliary but not core cellular functions. They are likely, or could have been during their evolutionary history, shuffled as part of their mobilomes. Hence, the evolutionary entanglement between the Asgard archaea and the Eukaryota must be understood in the pan-Asgard space and in the context of genome size expansion.

Thus, our analyses collectively suggest a plausible scenario where an ancestral Heimdall group archaeon with a small genome engaged in endosymbiosis with a bacterium and established the archaeal basis of information processing in the first eukaryotic common ancestor (FECA). The remaining defining features of eukaryotes are a result of decentralized innovations across the tree of life that became hierarchically imported, most frequently and often indirectly, through Asgard archaea lineages closest to FECA, to ultimately orchestrate LECA (Fig. 6c). As such, it is possible that the acquired non-essential genes were later co-opted to serve essential functions as the archaeon–bacterium symbiont expanded its regulatory complexity. We refer to this conceptual framework as the Heimdall nucleation–decentralized innovation–hierarchical import (HDH) model for future implementation and debate.

## Discussion

The contiguous and complete genomes of Asgard archaea constructed in this study allowed us to resolve the composite origins of their genetic repertoires and identify diverse, unique mobile elements as their drivers. One important facet to be considered is timescale. While the pivotal role of horizontal transfer in the diversification of Asgard archaea is evidenced by the high number of bacteria-related genes found in this study, a considerable fraction of these genes is likely now stable in their respective lineages and only a certain fraction is a part of their present-day mobilomes—the entire set of mobile elements in a genome. However, the uncharted features, such as the extraordinarily large proteins in aloposons and Asgard-specific host range of mobile elements found in this study, suggest that the Asgard archaea mobilome may still hold ancient signatures inherited around the time of eukaryogenesis. Expanding the repertoire of complete genomes in a broader Asgard archaea taxonomic range, pan-genomic analyses of the same or closely related species and molecular clock approaches will together help chronicle the horizontal transfer events across their evolutionary history. Given that the presence of bacterial genes is prevalent in both branches of the Asgard–eukaryote sisterhood, it will be particularly exciting to explore the extent to which bacterial genes have been transferred into their shared ancestors before eukaryogenesis.

Genome size variability in both eukaryotes and prokaryotes have been attributed to rapid expansion driven by mobile elements followed by gradual erosion under natural selection (such as nutrient availability)^45,46^. It is thus reasonable to assume that such expansion–erosion cycles would have occurred around the time of eukaryogenesis. While the mechanism of genome expansion around eukaryogenesis is genetic, which will be further elucidated by future discoveries of more Asgard archaea mobile elements, the selection pressure for these traits is ecophysiological. In this study, we showed that the influx of genes into the Asgard archaea is highly constrained by genome size in a similar fashion as in eukaryotes. Hence, resolving the ecophysiological drivers of genome size stratification across Asgard archaea lineages may help us unlock the origin of eukaryotic genome complexity.

### Etymology

#### *Ca. H. endolithica* PR6

Heimdall, watchman of the gods in Norse mythology; archaios (Greek), ancient, primitive; endo- (Greek), within; lithos (Greek), rock). Proposed classification: class Ca. Heimdallarchaeia, order Ca. Heimdallarchaeales, family Ca. Heimdallarchaeaceae, genus *Ca. Heimdallarchaeum*.

#### *Ca. H. aukensis* PM71

Heimdall, watchman of the gods in Norse mythology; archaios (Greek), ancient, primitive; Auka, the local hydrothermal vent field in the southern Pescadero Basin where the species originated; -sis (Greek), process or condition. Proposed classification same as above.

#### *Ca. H. repetitus* FW102

Harpocrates, Greek god of silence; archaios (Greek), ancient, primitive; repetita (Latin), repetitive (referring to the high fraction of repetitive sequences that constitute 4% of the genome). Proposed classification: class Ca. Lokiarchaeia, order Ca. Lokiarchaeales, family Ca. Prometheoarchaeaceae, genus *Ca. Harpocratesius*.

## Methods

### Hydrothermal vent rock and sediment sample collection

Rock no. NA091-R045 (source of *Ca. H. endolithica* PR6, *Ca.* *H. repetitus* FW102 and Thorarchaeote FW25) and rock no. NA091-R008 (source of Heimdall group Gerdarchaeote AC18) were retrieved from the Auka hydrothermal vent site situated on the margin of the southern Pescadero Basin of the Gulf of California using remotely operated vehicle *Hercules* during research expedition NA091 on *E/V Nautilus* on 2 November 2017. Local venting fluids have a measured temperature approaching 300 °C, contain hydrocarbons and hydrogen and are precipitating minerals, such as calcite and barite^15^. R045 was collected during dive H1658 at coordinates 23.956987786° N, 108.86227922° W at a water depth of 3,674 m, near shimmering water, a sign of locally focused hydrothermal fluid discharge. R008 was collected during dive H1657 at coordinates 23° 57′ N, 108° 52′ W at a water depth of 3,651 m. After shipboard recovery, rock samples were placed in Mylar bags prefilled with 0.2 µm filtered bottom seawater collected during the same dive, flushed with N_2_ gas for 10 min, sealed and stored at 4 °C until preparation for incubations in the laboratory.

Sediment sample no. FK181031-S0193-PC3 (source of *Ca. H. aukensis*) was collected during the research expedition FK181031 on *R/V Falkor* to the southern Pescadero Basin on 14 November 2018. The sample was collected during dive S193 at the Auka hydrothermal vent site (23.954822° N, 108.863009° W, water depth of 3,657 m), near the site where rocks nos. NA091-R045 and NA091-R008 were collected in 2017. The sediment push core was extruded upwards and sectioned into discrete 3 cm depth horizons on board immediately after recovery, transferred into sterile Whirl-Pak bags and sealed in a larger Mylar bag, flushed with argon gas, heat-sealed and stored at 4 °C until use in the laboratory.

Sample collection permits for the expedition were granted by the Dirección General de Ordenamiento Pesquero y Acuícola, Comisión Nacional de Acuacultura y Pesca (Permiso de Pesca de Fomento no. PPFE/DGOPA-200/18) and the Dirección General de Geografía y Medio Ambiente, Instituto Nacional de Estadística y Geografía (authorization no. EG0122018), with the associated diplomatic note no. 18-2083 (CTC/07345/18) from the Secretaría de Relaciones Exteriores-Agencia Mexicana de Cooperación Internacional para el Desarrollo/Dirección General de Cooperación Técnica y Científica.

### Artificial seawater medium recipe

Artificial seawater was prepared as described in Scheller et al.^47^ with minor modifications. Briefly, 1 l of artificial seawater (ASW) medium contained 46.6 mM MgCl_2_, 9.2 mM CaCl_2_, 485 mM NaCl, 7 mM KCl, 20 mM Na_2_SO_4_, 1 mM K_2_HPO_4_, 2 mM NH_4_Cl, 1 ml of 1,000× trace element solution, 1 ml of 1,000× vitamin solution and 0.5 mg of resazurin and was buffered by 25 mM HEPES buffer adjusted to pH 7.5. One litre of 1,000× trace element solution contained 50 mM nitrilotriacetic acid, 5 mM FeCl_3_, 2.5 mM MnCl_2_, 1.3 mM CoCl_2_, 1.5 mM ZnCl_2_, 0.32 mM H_3_BO_3_, 0.38 mM NiCl_2_, 0.03 mM Na_2_SeO_3_, 0.01 mM CuCl_2_, 0.21 mM Na_2_MoO_4_ and 0.02 mM Na_2_WO_4_. One litre of 1,000× vitamin solution contained 82 μM d-biotin, 45 μM folic acid, 490 μM pyridoxine, 150 μM thiamine, 410 μM nicotinic acid, 210 μM pantothenic acid, 310 μM para-aminobenzoic acid, 240 μM lipoic acid, 14 μM choline chloride and 7.4 μM vitamin B_12_.

### Enrichment cultivation

Rock no. NA091-R045 was anaerobically fragmented; then, approximately 5 g wet weight was crushed using a sterile agate mortar and pestle on 8 November 2018 and immediately immersed in anaerobic ASW medium in 25–125 ml of butyl rubber-stoppered serum bottles supplemented with different carbon/energy sources, including lactate, H_2_/CO_2_, hexane and decane and incubated in the dark at 40 °C (Extended Data Fig. 1a). The headspace for all cultures was flushed and overpressurized with N_2_ gas (2 atm). For the H_2_-containing cultures, the N_2_ gas headspace was replaced with H_2_/CO_2_ at an 80:20 mixture by flushing for 1 min and subsequent equilibration at 2 atm. After 33 d of incubation, the lactate-fed first-generation culture produced 5 mM sulphide, indicating active sulphate reduction. This enrichment was mixed by gentle shaking and diluted 1:100 vol/vol into fresh anaerobic ASW medium containing the same suite of carbon/energy sources as described above (Extended Data Fig. 1b). A transfer using the liquid fraction-lacking rock particles from the primary lactate enrichment was also included to enrich for members of the planktonic community alone with lactate as the carbon and energy source. This enrichment was later found to be devoid of the AAG (Heimdall) phylotype. Third- and fourth-generation cultures were set up in the following months through 1:100 dilution (Extended Data Fig. 1b). Further details of microbial community development in these enrichments are provided in Supplementary Note 1 and Supplementary Tables 1–3.

R008 was prepared as above except using 2 atm of methane in the headspace as the sole carbon source and electron donor. The culture was passaged twice using a 1:100 dilution under the same culturing conditions; the cell fraction was collected by centrifugation after a total of 22 months for metagenomic sequencing (described below).

For sediment enrichment cultivation, the top 3 cm section of the sediment core was mixed with anaerobic ASW at a 1:4 vol/vol ratio; a total of 60 ml volume each was dispensed into seven 125 ml glass serum bottles sealed with butyl rubber stoppers. The headspace was replaced by ethane (2 atm) in 2 bottles (Supplementary Table 5), while the headspace in 1 bottle was replaced by 100% N_2_ gas (2 atm). The cultures were incubated at 37 °C in the dark. Further details on microbial community development are provided in Supplementary Note 1 and Supplementary Table 4.

### Mineralogical analyses

The mineralogical composition of rocks NA091-R045 and R008 was characterized on a PANalytical X’Pert Pro X-Ray diffractometer. A dried rock aliquot was finely powdered using a clean agate mortar and pestle and scanned from 3 to 75° (2*θ* angle) at a 0.0167° step size. Mineral identification was performed with the X’Pert HighScore software v4.1 using the search and march algorithm.

### DNA extraction

Combined cells with rock or sediment substrate were pelleted through centrifugation at 13,000 r.p.m. for 3 min. For amplicon sequencing, unless specified in Supplementary Table 6, DNA was extracted using the Qiagen DNeasy PowerSoil kit (catalogue no. 47014) according to the manufacturer’s instructions as described previously^48^ with a minor modification, where mechanical shearing was carried out using the MP Biomedicals FastPrep-24 system (catalogue no. 116004500) at level 5.5 for 45 s. For genomic sequencing, incubated rock and sediment cultures were extracted using multiple approaches, including the Qiagen DNeasy PowerSoil kit, ZymoBIOMICS 96 MagBead DNA Kit (catalogue no. D4302; Zymo Research Corporation), *Quick*-DNA 96 Kit (catalogue no. D3010; Zymo Research Corporation), ZymoBIOMICS DNA Microprep Kit (catalogue no. D4301; Zymo Research Corporation) and a standard phenol/chloroform-based protocol. The list of samples and their extraction methods are provided in Supplementary Table 6.

### 16S rRNA gene amplicon sequencing

For amplicon (iTAG) sequencing of 16S rRNA genes, extracted DNA was amplified using primer pair 515f/806r GTGCCAGCMGCCGCGGTAA/ GGACTACHVGGGTWTCTAAT, barcoded and sequenced at Laragen using the Illumina MiSeq platform and analysed using Qiime v.1.8.0 (ref. ^49^) as described previously^48^. Taxonomic assignment was based on the SILVA 138 database (https://www.arb-silva.de)^50^.

Full-length 16S archaeal rRNA gene sequences were amplified using the archaeal primer pair SSU1Arf/SSU1492Rngs TCCGGTTGATCCYGCBRG/ CGGNTACCTTGTKACGAC as described by Bahram et al.^51^, multiplexed as instructed by PacBio and sequenced using the PacBio Sequel II at the Brigham Young University DNA Sequencing Center and then analysed using the DADA2 package v1.9.1 in R v3.6.0 as described in Callahan et al.^52^ using the SILVA 138 database for taxonomic classification. Note that in the SILVA 138 database, all Asgard archaea clades are classified under Asgardarchaeota.

### Metagenomic sequencing

A total of 11 metagenomic sequencing runs were performed using the Illumina and Oxford Nanopore platforms, with details listed in Supplementary Table 6. For Illumina short-read sequencing, libraries were constructed using the NEBNext Ultra and Nextera Flex Library kits as specified in the Supplementary Table 6. Sequencing was carried out using a HiSeq 2500 system (single-end, 100 bp) at the Caltech Genetics and Genomics Laboratory and HiSeq 4000 system at Novogene (paired-end, 150 bp). Only paired-end data were used for assembly, while all data were used for error correction. Due to the low DNA quantity obtained from the sediment incubation that yielded *Ca. H. aukensis*, we used multiple displacement amplification with the QIAGEN REPLI g Midi Kit before library preparation for Nanopore sequencing. Oxford Nanopore sequencing libraries were constructed using the PCR Barcoding Kit (catalogue no. SQK-PBK004) and were sequenced on MinION flow cells FLO-MIN106. Base calling was performed with the ONT Guppy software v.3.4.5.

### Genome assembly, error correction and read coverage mapping

Two different approaches were used to assemble contiguous genomes from metagenomes. For species of interest, if Nanopore sequencing yielded high read coverage and read lengths N50 > 2 kb, we obtained more contiguous genomes through de novo assembly purely based on Nanopore reads. If Nanopore sequencing did not yield a high number of reads or exhibited low read lengths, we obtained more contiguous genomes through de novo assembly first based on Illumina reads and then joined using Nanopore reads.

For *Ca. H. endolithica*, Nanopore sequencing data were assembled de novo using Canu^17^ v.2.1, which yielded a 30 Mbp assembly, including a 3.4 Mbp contig. The approximate 40 kilobase (kb) regions at two ends of an approximate 3.4 Mbp contig were repetitive. This repeated region was deleted at one end and the two ends were joined to result in a circular genome. The resulting genome was mapped using BamM (http://ecogenomics.github.io/BamM/, based on Burrows–Wheeler Aligner^53^ mapping) with 150 bp Illumina paired-end reads (88× coverage on average) and 100 bp single-end reads (20× coverage). Mapped reads were then used for error correction through pilon^54^ v.1.22. To account for the reduced mapping at the edges (approximate 50 bp region), the two ends of the genomic sequence were joined, read-mapped and error-corrected again using the same methods. After the genome was annotated, it was rotated such that the genomic sequence ended with tRNA (GlyCCC), which was the integration site of the putative provirus HeimV1. All sequencing reads derived from incubations of the same rock were mapped onto the final genome using BamM, which was then used for coverage calculation through bedtools (https://bedtools.readthedocs.io/en/latest/).

For *Ca. H. aukensis*, Illumina PE150 bp sequencing data were assembled using SPAdes^18^ v.3.14.1 with the ‘-meta’ option and *k*-mers 21,33,55,77,99. The assembly was then scaffolded using Nanopore reads through two iterations of LRScaf^55^ v.1.1.10. The *Ca. H. aukensis* genome was joined after trimming the identical sequences at the two ends. The end-joining region was verified through PCR amplification and Sanger sequencing using the primer pair CGCTTTCTTCAAACAATATTTCTGGTG/CTTACTTTCTCTCGGTCCATTTTTCAC. Finally, a 1 kbp stretch of unresolved genomic sequence at an approximate 2.9 Mbp position was resequenced through PCR amplification and Sanger sequencing using the primers GAGTTTTTTCAATCTTATAATGCCAAACTAAAAAATAG (forward), CAGTCAGATTTGACACAATTTTGGTC (reverse) and GCTGGACTCAACCTATAACTAATAGT (reverse). The final assembly was read-mapped, error-corrected through pilon v.1.24 using 346× coverage. It was rotated as described above to place the tRNA gene GlyCCC at the end.

The metagenome containing the Lokiarchaeote *Ca. H. repetitus* FW102 was assembled using Canu v.2.1, as described for the *Ca. H. endolithica* genome, and then binned using metabat2 v.2.15 (ref. ^56^) with default parameters. The bin was then used to recruit long reads using minimap2 v.2.17 and reassembled and binned again. We then used LRScaf to scaffold the contigs and used ten iterations of pilon v.1.24 to achieve error correction and resolve ambiguous bases.

The Thorarcheote FW25 MAG was assembled using the hybrid assembly of Illumina reads and Nanopore reads using SPAdes v.3.14.1 with *k*-mers 21,33,55,77,99, and then binned using metabat2 v.2.15 with default parameters. The MAG bin was then used to recruit reads through MIRAbait in the MIRA v.4 package (http://mira-assembler.sourceforge.net/docs/DefinitiveGuideToMIRA.html#chap_intro). These reads were then used for hybrid assembly with Nanopore long reads via SPAdes v.3.14.1 with *k*-mers 21,33,55,77,99. It was then binned again using metabat2 v.2.15 with default parameters to yield the final Thorarcheote FW25 MAG.

The metagenome containing Gerdarchaeote AC18 was assembled from Illumina reads using SPAdes v.3.14.1 with *k*-mers 21,33,55,77,99 and then binned using metabat2 v.2.15 with default parameters. The MAG bin was then used to recruit reads through MIRAbait in the MIRA v.4 package and then reassembled and binned using SPAdes and metabat2 to yield the final Gerdarchaeote AC18 bin.

### Alignment fraction, ANI and AAI

ANI and alignment fraction values, independently calculated for rRNA, tRNA and coding gene sequences were obtained using ANIcalculator^57^ 2014-127, v.1.0 (https://ani.jgi.doe.gov/html/download.php?). Note that Lokiarchaeote FW102 contains 2 copies of 16S rRNA genes at 99% identity with each other, and Thorarchaeote BC has a partial 16S rRNA gene. The alignment of 16S rRNA was carried out using SINA^58^ v.1.2.11. The AAI values of translated proteomes were obtained with the enveomics package v1.8.0^59^. The final output is shown in Supplementary Table 7.

### Genome and mobilome annotations

Gene calling was done using a combination of Prodigal v.2.6.3 and Glimmer v.3.0.2 using translation code 11 within the RASTtk^60^ pipeline, now under the PATRIC package v1.032^61^. Translated coding sequences were annotated and domain-assigned using eggNOG mapper^39^ v.2. The tRNA, 16S rRNA and 23S rRNA genes were identified using RNAmmer^62^ v.1.2 embedded in RASTtk. Thus far, 5S rRNA gene sequences could not be predicted through the existing HMM using various approaches. Long, non-tandem repeats were identified using RASTtk with the default cut-off of 95% identity and 100 bp. Tandem repeat sequences were identified using RASTtk, Prokka v1.14.6 and CRISPRCasTyper 1.1.4^63^. Prokka and CRISPRCasTyper both employ MinCED (https://github.com/ctSkennerton/minced) to identify repeats and detect intragenic tandem repeats, which were manually removed from the CRISPR–Cas analyses. The Cas genes were annotated using CRISRCasTyper.

All identified *Heimdallarchaeum* mobilomes were further analysed using PSI-BLAST 1.10.0^64^, CDD search v3.19^65^ and PhANNs webserver (version March 2021)^37^.

### Genome evaluation and HMM construction

Marker coverage was carried out using a two-step process. First, we used the automated marker analyses via CheckM^66^ v.1.1.3 with the lineage_wf option and the default HMM *E* value cut-off, which included the 149 standard archaeal single-copy marker set. Next, each of the missing markers was examined with hmmer^67^ v.3.3.2 using the hmmsearch option with manual inspection of alignment regions and bitscores. This rescued markers unidentified through the default cut-offs by CheckM as well as divergent variants that most likely functionally replace the genuinely missing marker. The detailed description of markers missed by CheckM can be found in Supplementary Note 2 and the final evaluation of marker presence is displayed in Extended Data Fig. 4a and Supplementary Table 15. Next, we constructed an updated HMM set to replace the CheckM set by (1) updating all HMM to the most recent versions, (2) removing the six commonly missing or duplicated markers shown in Extended Data Fig. 4a from the list and (3) overcoming the pitfall of existing HMMs constructed using only a few sequences acquired from Euryarchaeota and Crenarchaeota. We manually constructed Asgard-specific versions based on the 282 Asgard archaea genomes. The HMMs constructed in this study are PF00832.ASG, PF00861.ASG, PF01194.ASG, PF01287.ASG, PF01667.ASG, PF03874.ASG, PF03876.ASG, PF13656.ASG, TIGR00270.ASG, TIGR00336.ASG, TIGR00442.ASG, TIGR02338.ASG and TIGR03677.ASG. The updated HMM file has been provided as a supplementary data file. The updated HMM was used to evaluate the 282 genomes reported in this study and in the literature^3,6–12,16,23,26,68–77^ through (1) CheckM, which uses Prodigal for gene calling, and (2) the more up to date HMMER3.2.2 on our gene calls described above. The latter generally produced slightly higher completeness and redundancy values (Supplementary Tables 8 and 9). For the expanded set of Asgard archaea genomes used for the phylogenomic analyses shown in Extended Data Fig. 4b, we applied the following filtering criteria: ≤100 contigs, >96% marker completeness and <8% marker redundancy. We also took the evenness of taxonomic sampling into account. The set is also shown in the Asgard archaea tree in Extended Data Fig. 2. The importance of genome quality evaluation is highlighted in Extended Data Fig. 9.

### Phylogenomics

A phylogenomic tree of Asgard archaea was constructed with IQ-TREE v.2.1.2 (ref. ^78^) using a partitioned analysis^79^ with model selection using ModelFinder^80^ and 1,000 ultrafast bootstrap replicates using UFBoot2^81^ on a concatenated alignment generated from MUSCLE^82^ v.3.8.1551 alignments of 76 archaeal marker genes identified in the genomes using HMMs included with anvi’o v.6.2 (ref. ^83^). The phylogenomic tree was visualized using iTOL^84^ and rooted with the TACK superphylum.

The Archaea–Eukaryota phylogenomic tree, including the Asgard genomes discussed in this study, was constructed based on the 56 Archaea–Eukaryota ribosomal proteins used by Zaremba-Niedzwiedzka et al.^3^ using reference sequences from the corresponding Dryad repository. In addition to the Asgard archaea identified in this study, additional sequences of the most complete genomes representing different lineages of the TACK superphylum were added to the dataset. Sequences of 56 archaeal COGs obtained from the Dryad repository were used as reference databases to retrieve homologous sequences from target genomes using BLAST^85^ v.2.10.1. Each set of archaeal COG sequences were aligned using MUSCLE v.3.8.1551 and inspected and trimmed manually. Manually trimmed alignments were then further trimmed using BMGE^86^, recoded to four-state SR4 using a custom script (https://github.com/dspeth/bioinfo_scripts/tree/master/phylogeny) and finally concatenated and converted to PHYLIP format using catfasta2phyml v1.1.0 (https://github.com/nylander/catfasta2phyml). The final concatenated, recoded alignment was used to calculate phylogenies using IQ-TREE v.2.1.2 (ref. ^78^) using a C60 model adapted for SR4 recoded data by Zaremba-Niedzwiedzka et al.^3^ and 1,000 ultrafast bootstrap replicates using UFBoot. The phylogenomic tree was visualized using iTOL^84^ and rooted with *Euryarchaeota* as the outgroup. The genomes and conserved genes used for the phylogenomic analyses are listed in Supplementary Tables 16 and 17.

### Discovery of *Heimdallarchaeum*-targeting mobile elements through CRISPR spacer targeting

Repeat sequences from the *Heimdallarchaeum* CRISPR arrays were used to blast against the CRISPR repeats we recruited, using CRISPRCasTyper, from multiple databases with a 95% alignment and 95% identity cut-off. The databases include GTDB v.95, our in-house assemblies from the Pescadero Basin (this study, F.W. et al. manuscript in preparation and Speth et al.^87^; Supplementary Table 10, 22 sets) and published assemblies from the Guaymas Basin^22^ (Supplementary Table 11, 16 sets).

While no homologous CRISPR repeats were found in the entire GTDB database, we found several CRISPR arrays from the Guaymas and Pescadero assemblies with identical repeats to the *Heimdallarchaeum* CRISPR repeats found in this study, demonstrating the specificity of the CRISPR discovery approach. Since both the Guaymas and Pescadero CRISPR sets comprise assembled sequences that were not de-replicated, the entire CRISPR spacer collection from the recruited CRISPR arrays was de-replicated using a 100% identity cut-off. Notably, no spacer overlap was found between the Guaymas and Pescadero CRISPR sets. In total, the final de-replicated, putative *Heimdallarchaeota* spacerome in this study consisted of 455 from the 2 original *Heimdallarchaeum* genomes, 578 from the Pescadero Basin assemblies and 532 from the Guaymas Basin assemblies. We note that the above set likely only represents a fraction of the true *Heimdallarchaeum* spacerome given that the original CRISPR repeats came from only two species.

Next, to identify potential mobile genetic elements (MGEs) targeted by the *Heimdallarchaeum* spacerome, we used BLAST to search for spacer matches in the above three assembly datasets, the two *Ca. Heimdallarchaeum* genomes and various published virus databases/datasets, which are the RefSeq virus database r98^88^, IMG/VR v.3 (ref. ^36^) and the huge phage^89^, giant virus^90^ and Loki’s castle virus datasets^91^. To avoid self-matches, the CRISPR arrays containing the spacers were replaced by Ns in their respective assemblies. For the homology cut-off, we used 95% alignment and 95% identity as described previously^92^. Strikingly, no spacer matches were found from any of the viral datasets or GTDB genome database. The spacer matches to the Guaymas and Pescadero Basins metagenomes are listed in Supplementary Tables 12 and 13.

We then de-replicated the putative MGEs/viruses identified above using BLAST, removed contigs smaller than 2.8 kb and manually examined the target gene neighbourhoods and potential self-match due to CRISPR arrays that evaded detection and blocking. These contigs, together with the ones described in Fig. 2, ultimately constitute the 56 putative Heimdallarchaeota MGEs listed in Supplementary Table 14.

### Resolution of the genomic insertion and circularization of HeimV1

To capture the two different states during the life cycles of HeimV1 (Fig. 3f), we used three primer sets to amplify the sequences around the two insertion sites of HeimV1 and confirmed them using gel electrophoresis and Sanger sequencing. Set 1 amplified the region between upstream tRNA GlyCCC in the *Ca. H. endolithica* genome and the first coding gene of the HeimV1 (GTGAATCAATAGCTTTCACTTATAATGAG/GTGATTGTATTAAGTCTGCAACATATTC). Set 2 amplified the regions containing the transposase in the *Ca. H. endolithica* genome and the integrase in HeimV1 (CTTAGATATGTACGTGATAGGATCATATG/CTTCTTTCCTCTTTTTGTCTCTGCTTC). Set 3 amplified the two ends of the circular HeimV1 (CTTAGATATGTACGTGATAGGATCATATG/GTGATTGTATTAAGTCTGCAACATATTC). Each primer set amplified approximately 2 kb of target regions with set 1 and set 2 indicating the presence of the integrated state of HeimV1 and set 3 indicating the circular state.

### Protein clustering of integrases and transposases

Protein sequences showing integrase and transposase domains, identified using eggNOG mapper from the 8 Asgard archaea MAGs, were pooled and clustered at 90% sequence identity using cd-hit^93^ v.4.8.1. The resulting representative sequences were used for two sequential rounds of homology searches using DIAMOND^94^ v.2.0.6 against the protein sequences obtained from the GTDB v.95 genome database. A cut-off of >20% sequence identity, >85% sequence alignment and <15% length difference was used for the first round; a cut-off of >30% sequence identity, >90% sequence alignment and <10% length difference was used for the second round. The resulting protein sequences were combined with the Asgard archaea integrases/transposases originally pooled and were clustered together using 95% sequence identity with cd-hit. The resulting 96,367 representative sequences were clustered using ASM-Clust^95^ with a sequence subset size of 5,000 to generate the alignment score matrix, using default values for the other settings.

### Taxonomic profiling through protein orthologues

The taxonomic clustering and COG analyses were carried out using eggNOG mapper^39^ with the eggNOG orthologue database v.5.0. The protein counts belonging to each taxonomic group (Archaea/Bacteria/Eukaryota/Unassigned) were extracted from the output and fitted linearly with MATLAB R2018a using the polyfit function and yielding Fig. 5c.

Since different proteins evolved at different rates, we combined the use of a single cut-off-based protein clustering approach with functional domain-based manual refinement to capture and compare ERPs across the lineages selected in this study. First, we used BLAST v.2.2.26 to evaluate the sequence homologies within the entire proteome of the eight MAGs in this study. We then used an 80% alignment length (relative to the length of the shorter protein sequence) and 0.24 alignment × identity cut-off to yield candidate protein clusters, which we then cross-referenced with the Eukaryota group in the eggNOG classification to generate 227 candidate ERP clusters. Finally, we manually examined the relatedness within and between each ERP cluster through batch searches using the conserved domain database^65^. This led to the recombination of the candidate ERP clusters into the functionally distinct 135 ERP families. To align with previous work^3,6^, all small GTPases were classified as one single ERP family, constituting 291 proteins from the 8 representative Asgard archaea MAGs.

### Maximum-likelihood analyses of proteins encoded by HeimV1 and HeimV2

Homology search for all peptide sequences of HeimV1 through DIAMOND^94^ v.2.0.6 was carried out against the GTDB v.95, Pescadero Basin and Guaymas assemblies, RefSeq virus database^88^, IMG/VR^36^ and huge phage^89^, giant virus^90^ and Loki’s castle virus datasets^91^. The search outputs were pre-clustered with a 70% identity cut-off using cd-hit v.4.8.1 (ref. ^93^). The representative sequences were aligned using the MAFFT v.7.475 (ref. ^96^) option linsi and trimmed with trimAl v.1.4.1 (ref. ^97^), option gappyout. Maximum-likelihood analyses were carried out with IQ-TREE v.2.1.12 (ref. ^78^) using the LG4X model and ultrafast bootstrap with 2,000 replicates. The phylogenetic tree was visualized and prepared using iTOL^84^.

### Reporting Summary

Further information on research design is available in the Nature Research Reporting Summary linked to this article.

## Supplementary information

Supplementary Information Supplementary Notes 1 and 2.

Reporting Summary

Peer Review Information

Supplementary Tables Supplementary Tables 1–17.

Supplementary Data 1 Hidden Markov Models for Asgard archaea markers.

Supplementary Data 2 Sequences of mobile elements targeting *Ca. Heimdallarchaeum* spp.

## Data Availability

The assembled genomes and raw metagenomic sequencing reads can be found on the National Center for Biotechnology Information database under BioProject no. PRJNA721962. Source data are provided with this paper.

## Source data

Source Data Fig. 1 Phylogenomic trees in Newick format.
Source Data Fig. 4 Phylogenetic trees in Newick format.
Source Data Fig. 5 Numerical data.
Source Data Fig. 6 Numerical data.
Source Data Extended Data Fig. 1 Numerical data.
Source Data Extended Data Fig. 2 Phylogenetic trees in Newick format.
Source Data Extended Data Fig. 4 Phylogenetic trees in Newick format.
Source Data Extended Data Fig. 5 Numerical data.
Source Data Extended Data Fig. 6 Numerical data.
Source Data Extended Data Fig. 8 Phylogenetic trees in Newick format.
Source Data Extended Data Fig. 9 Numerical data.

## Code availability

The custom script for recoding of amino acid sequences to four-state SR4 can be found at https://github.com/dspeth/bioinfo_scripts/tree/master/phylogeny. Other custom scripts can be found at https://github.com/wufabai/genomics.
