# Supplementary material for: Unique mobile elements and scalable gene flow at the prokaryote–eukaryote boundary revealed by circularized Asgard archaea genomes
Source: Nat Microbiol. 2022 Jan 13;7(2):200–12. doi: 10.1038/s41564-021-01039-y (PMC8813620; doi:10.1038/s41564-021-01039-y)
Supplement: Supplementary file 1 — Supplementary Notes 1 and 2. [file 41564_2021_1039_MOESM1_ESM.pdf]

nature portfolio

https://doi.org/10.1038/s41564-021-01039-y

# Supplementary information

# Unique mobile elements and scalable gene flow at the prokaryote–eukaryote boundary revealed by circularized Asgard archaea genomes

In the format provided by the authors and unedited

Supplementary information for

# Unique mobile elements and scalable gene flow at the prokaryote-eukaryote boundary revealed by circularized Asgard archaea genomes

Fabai Wu[1,2,#], Daan R. Speth[1,2], Alon Philosof[1], Antoine Crémière[1], Aditi Narayanan[2], Roman A. Barco[3], Stephanie A. Connon[1], Jan P. Amend[3,4], Igor A. Antoshechkin[2] & Victoria J. Orphan[1,2,#]

[1]Division of Geological and Planetary Sciences, California Institute of Technology, Pasadena, CA, 91125, USA

[2]Division of Biology and Biological Engineering, California Institute of Technology, Pasadena, CA 91125, USA.

[3]Department of Earth Sciences, University of Southern California, Los Angeles, CA, USA.

[4]Department of Biological Sciences, University of Southern California, Los Angeles, CA, USA.

[#]Correspondence: wu.fa.bai@gmail.com; vorphan@gps.caltech.edu

## Supplementary Notes

### 1. Enrichment of *Ca. H. endolithica* and *Ca. H. aukensis* from rock- and sediment-hosted microbial assemblages

The original rock was dominated by methane-oxidizing archaea belonging to the ANME-1 clade (Supplementary Table S1). Besides methane, probing of hydrothermal fluid at the site of collection also indicated the existence of hydrocarbon and $H_2$[1], indicating complex energy sources supporting the microbial community. The series of incubations described in this study was aimed at exploring the metabolic potential of the non-ANME members of the community, using medium-chain hydrocarbon (decane) and $H_2$, as well as common metabolic intermediates - lactate and acetate. Sulfate was supplied at 20mM as electron acceptor. The changes in the relative abundance of *Ca. H. endolithica* in various conditions are shown in Extended Data Fig. 1, and all amplicon sequencing data of lactate-related passages are in Supplementary Table S1.

Members of the initially characterized community have varying levels of association with the rock. To qualitatively examine such associations, we gently sonicated and rinsed the rock fragments and carried out DNA extraction and amplicon sequencing with the remaining solid-phase sample, we observed drastic differences. ANME-1 and its putative sulfate-reducing partner, *Desulfofervidus sp.*, respectively showed higher relative abundance in the rock after sonication from 41 to 50% and from 3% to 17%, indicating an endolithic lifestyle. On other hand, three most abundant bacterial clades belonging to Campylobacterales, Bacteriodetes, and Sulfurimoas were depleted from the rock by the treatment, reducing from 3-6% to below 0.1%, indicating that they are not endolithic. AAG clade increased from undetecSupplementary Table to 0.1% after sonication. The endolithic lifestyle of AAG was further demonstrated by the fact that this group was diluted out through pure planktonic passage in culture (Extended Data Fig. 1c, S1d, Supplementary Table S1). Indeed, paraformaldehyde-fixed, post-incubation rocks showed formation of large, multispecies biofilms, although confident detection of the AAG-associated cells has not yet been ascertained (Extended Data Fig. 1g).

The 1$^{st}$- and 2$^{nd}$-generation lactate-supplemented culture produced up to 16 mM sulfide during the course of incubation (Supplementary Table S2). This was correlated with the dominance of sulfate-reducing taxa, *Desulfohalobiaceae* (family), *Desulfocapsaceae* (family), and *Desulfarculaceae* (family) (Supplementary Table S1). These clades were found in both rock-associated and planktonic passages. As indicated by 16S rRNA gene amplicon sequencing, other taxa that existed above 1% of the communities are *Thermovirga* (genus), *Marinitoga* (genus), and *Marinimicrobia SAR406 clade* (phylum). While the 16S rDNA sequence of AAG was below detection limit (~0.005%) in the original inoculum via amplicon sequencing, the relative abundance of this group started increasing after 120 days of incubation at 40°C. By 200 days, the relative abundance of AAG reached 1.8% in the 2$^{nd}$-generation culture and was later detected at 3.6% and 1.0% in the associated 1$^{st}$- and 3$^{rd}$-generation transfer cultures after 1 year (Extended Data Figs.1a-1d, Supplementary Table S1). These resulting AAG relative abundance in the lactate-supplemented cultures was substantially higher than cultures from the same rock with other energy/carbon substrates (Extended Data Figs. 1b, 1c).

Drastic reduction in community complexity was observed via two indexes: 1) number of OTUs above 0.005% (or 1 in 20000), and 2) Shannon diversity index (Extended Data Fig. 1e). In addition, full-length 16S rRNA gene sequencing further ascertained that the several archaea of interest existed in the 2$^{nd}$-generation lactate-supplemented culture without apparent close relatives at least at the 16S sequence level (Extended Data Fig. 1f, and Supplementary Table S3).

The sediment sample was initially dominated by methane-oxidizing archaea belonging to the ANME-2c clade and their sulfate reducing partner belonging to the *Dissulfuribacteraceae* family (Supplementary Table S4). The AAG phylotype was found to be 0.03% in the initial inoculum. After 9 months of incubation in artificial sea water (with $N_2$ or ethane headspace), the ANME-2c/*Dissulfuribacteraceae* partnership

became undetecSupplementary Table while various bacteria and AAG became dominant. The most abundant taxon in both ethane-containing incubations was Kosmotoga (genus, 7.9% and 13.88%), whereas AAG was ranked 3$^{rd}$ and 4$^{th}$, at 6.7% and 5.4% relative abundance. More than 20 taxa are above 1% in relative abundance in both cultures (Supplementary Table S4), which are substantially more complex than the incubated rock community described above. The two ethane-containing cultures produced approximately 1.3mM sulfide in the first three months, while the control culture produced 1.7mM sulfide, indicating that ethane did not stimulate sulfate-reducing activity (Supplementary Table S5). The increase in AAG relative abundance was also likely independent of ethane, since the control incubation with $N_2$ instead of ethane headspace showed 3.6% AAG after 9 months of incubation (Supplementary Table S4).

## 2. Detailed Marker evaluation of contiguous, complete genomes of Asgard Archaea representatives in this study.

CheckM-based marker analysis indicated 119 markers as single-copy across Asgard Archaea, 5 markers commonly missing, and 1 marker duplicated. Because the evaluation of presence/absence patterns based on Hidden Markov Models (HMMs) could vary due to the number of sequences they have built upon and the cutoff being applied, we manually inspected the missing markers in our circular genomes of *Ca. Heimdallarchaeum spp.* and previously reported circular genome of *Ca. P. syntrophicum*. We found that indeed the above commonly missing markers reported by CheckM are absent. However, we also found various markers reported missing in individual lineages do exist in these genomes, whose close homologs in the other Asgard Archaea MAGs were successfully identified as markers. There were 2 main factors leading to these missing annotations: 1) an outdated HMM that was built upon very few representative sequences, and 2) very short peptides such as the subunits of ribosomes and RNA polymerase that are very sensitive to homology cutoff and gene calling error. Below, we list the distribution and characteristics of individual markers.

**PF0832** is an HMM for the ribosomal protein L39e constituting 42 a.a. In CheckM report (containing version PF0832.15), this marker was missing in *Ca. H. aukensis*, Loki FW102, and Thor BC. However, through hmmsearch using an updated version PF0832.22, we found that all Asgard Archaea representatives encode L39e at a conserved location, next to *rpl31e* gene. We further constructed a new HMM PF0832.ASG for automated analyses.

**TIGR00442** is an HMM for histidine-tRNA ligase (hisS). In CheckM report, this marker was missing in *Ca. H. aukensis*. However, through hmmsearch, we found that *Ca. H. aukensis* contains a hisS gene highly homologous to the hisS in *Ca. H. endolithica* identified by CheckM. Hmmsearch also confirmed the single-copy hisS across examined genomes. We further constructed a new HMM TIGR00442.ASG for automated analyses.

**TIGR00432** is an HMM for an archaea-specific tRNA-guanine(15) transglycosylase (tgtA). CheckM included the version of this HMM from release version 15.0 (year 2014), which used only 3 sequences to build a 637 a.a. long HMM. Majority of its homologs in Asgard Archaea (except for Thorarchaeota) as well as other members of Euryarchaota and TACK are shorter, lacking the C terminal region. In comparison, the TIGR00432.2 version (updated by NCBI as part of PGAP) was built on 60 sequences and contain 441 a.a. In CheckM report, this marker was missing in *Ca. H. aukensis* and *Ca. H. endolithica*. However, using hmmsearch based on the updated TIGR00432.2, we found a single copy of archaeal tgtA in both genomes that contain sequences spanning the entire HMM.

**TIGR03683** is an HMM for the family of archaea-specific alanyl-tRNA synthetases, which contain a secondary additional doman (SAD). In CheckM report, this marker was missing in both Lokiarchaeotes and both Thorarchaeotes. We found that the two Lokiarchaeotes each encode a single copy of this enzyme (e-value <e-217). Their sizes are larger (998/1000 a.a. compared to the ~912 a.a. HMM), both containing an insertion around HMM position 501-529 and a duplication around HMM position 745-783. All Loki/Heimdall/Gerd versions also show a ~25 a.a. extension at the N terminus. Thorarchaeotes do not contain the full-length genes of this AlaS enzyme but do each encode a divergent AlaS that span most of

the HMM except for the a.a. positions around 1-56, 483-590, and 731-901. Notably, all genomes here except for Odin also encode additional proteins that span the C-terminal region of the full archaeal AlaS.

**PF13685** is an HMM for Iron-containing alcohol dehydrogenase Fe-ADH_2 -like protein. In CheckM report (version PF13685.1) it was missing in both Lokiarchaeotes genomes and Gerd AC18. We used hmmsearch based on the updated version PF13685.8, and indeed found that only Ca. *Heimdallarcheum spp*, Thorarchaeotes, and Odin LCB_4 encode this archaeal protein, which is also related to Glycerol-1-phosphate dehydrogenase (CD08174/COG0371). These proteins are closely related to those encoded by Bathyarchaeota. Lokiarchaeotes and Gerd AC18 encode paralogs that are more closely related to bacterial iron-containing alcohol dehydrogenase.

**TIGR00270** is an HMM for a conserved hypothetical protein. In CheckM report, it was missing in FW102 but present in *Ca. P. syntrophicum*. Hmmsearch identied a TIGR00270 protein in FW102, which has its closest homolog (through protein blast on the NCBI database) encoded by *Ca. P. syntrophicum* (90% alignment, 52% identity). The latter was identified by CheckM as a TIGR00270 family protein. Hmmsearch indicated that these two proteins are divergent from their homologs encoded by other Asgard Archaea. They only align with amino acid range 2-133 with the HMM, shorter than the range 2-154 covered by others. Given that this HMM was only constructed using 3 sequences from Euryarchaeota, the divergence of these sequences in the distant archaea lineages is reasonably expected. We further constructed a new HMM TIGR00270.ASG for automated analyses.

**TIGR03677** is an HMM for ribosomal protein L7ae. While in the CheckM report it was missing in *Ca. P. syntrophicum*, it was found through hmmsearch. As in all other Asgard Archaea genomes examined in this study, this L7ae-encoding gene (rpl7ae) is located immediately next to rps8ae gene. We further constructed a new HMM TIGR03677.ASG for automated analyses.

**PF13656** is an HMM for RNA polymerase Rpb3/Rpb11 dimerization domain (RNA_pol_L2). In the CheckM report (with version PF13656.1), it was missing in *Ca. P. syntrophicum*. Hmmsearch (using the new version PF13656.8) combined with CD-search and blastp indicate that Lokiarchaeotes pervasively encode an RNA_pol_L2 domain that is attached to a noncanonical N-terminal extension, but the sequence of this extension in different genomes is highly variable. We further constructed a new HMM PF13656.ASG for automated analyses.

**PF01194** is an HMM for RNA polymerase subunit N (polN). It was missing in the CheckM report (with version PF01194.12) for *Ca. P. syntrophicum*, possibly due to a failed gene calling. Our gene calling process only yielded a gene containing the C-terminal 42 a.a. PolN of *Ca. P. syntrophicum* can be found on the NCBI database with accession number WP_147665300.1, a 65 a.a. long protein containing the 42 a.a. protein above, indicating that the gene calling was due to a different site for the start codon. Hmmsearch using the current version of the HMM (PF01194.19) confirmed that WP_147665300.1 belongs to the PolN family. We further constructed a new HMM PF01194.ASG for automated analyses.

**PF01667** is an HMM for ribosomal protein S27e. In CheckM report (with version PF01667.12), it appeared missing in Thor FW25 and Gerd AC18. However, using an updated PF01667.19, they were all found to contain a single copy of S27e.

**TIGR00336** is an HMM for the pyrE-encoed orotate phosphoribosyltransferase. In CheckM report, it appeared missing in Thor BC alone but was present in Thor FW25. However, we found that the Thor BC encodes a PyrE that is highly homologous to the Thor FW25 PyrE. They both cover the whole HMM length but have a longer N-terminal extension (24 a.a.) than their homologs from other Asgard Archaea (10 a.a.). We further constructed a new HMM TIGR00336.ASG for automated analyses.

**TIGR02338** is an HMM for prefoldin subunit beta (PfdB). In CheckM report, it appeared missing in Thor BC alone but is present in Thor FW25. However, using hmmsearch, we found a single copy of Thor BC PfdB that is most homologous to the single-copy PfdB from Thor FW25 (94% alignment and 40% identity) and other Thorarchaeotes. While the Thor FW25 PfdB spans the HMM throughout, the first 9 a.a. of the 110 a.a.-long HMM was not aligned to Thor BC PfdB by hmmsearch. On the other hand, this N-terminal regions between these two Thor PfdB proteins are highly conserved. TIGR02338 was built using only 9

proteins, which likely caused alignment sensitivity to divergent sequences. We further constructed a new HMM TIGR02338.ASG for automated analyses.

In summary, besides the commonly missing 5 markers across the genomes, via cross-asgard and within-lineage comparisons, all genomes are found to contain all the expected markers with lineage-specific absence and duplications. In addition to the *Ca. Heimdallarchaeum spp.* detailed in the main text, we summarize the basic characteristics and marker coverage in the remaining genomes below.

**Lokiarchaeote FW102** contains 12 contigs, from the longest 966,990 bp to the shortest 5,127 bp. FW102 and the previously reported circular genomes ***Ca. Prometheoarchaeum syntrophicum***[2] show 70.2% AF and 17.5% ANI, indicating a relatedness at the family level. As indicated in Extended Data Fig.2e, these two genomes form a distinct branch more basal to the originally reported Loki clade discovered in Loki's Castle, which include a recently constructed, highly contiguous MAG Loki L04 (Caceres et al, BioRxiv 2019[3]), although the latter was less complete as evaluated by marker coverage and was not included in our analyses. In addition to the 5 shared with all Asgard Archaea representatives, both genomes lack 1 single-copy archaeal markers in this study, which is **PF13685.8** (Fe-ADH_2 Iron-containing alcohol dehydrogenase). They also both contain 2 duplications in the Asgard Archaea markers, which are TIGR00134 (GatD) and TIGR2153 (GatE), the D and E subunits of glutamyl-tRNA(Gln) amidotransferase. FW102 has 1 additional duplication (PF00398.15). In comparison, *Ca. P. syntrophicum* has 2 additional duplications (PF01864.12/CarS and PF00958.17/GMP_synt_C) and 2 additional triplications (PF00867.13/XPG_1 and PF00752.12/XPG_N).

**Thorarchaeote FW25** was assembled from the same sequencing data obtained for *Ca. H. endolithica*, although it was undetected in the amplicon sequencing due to the 16S primer bias against Thorarchaeota. It contains 15 contigs, ranging from the longest 1,553,122 bp and the shortest 5,353 bp. FW25 and previously reported **Thorarchaeote BC**[4] show 68.56% AF and 17.5% ANI, indicating a relatedness at the family level. Both genomes lack 1 single-copy archaeal marker **TIGR03683** (alanine tRNA ligase AlaS), which is likely functionally replaced by the smaller AlaS as described above that is unique to Thorarchaeotes. They also both contain 4 duplications in the Asgard Archaea markers, which are TIGR2153 (GatE), PF00867.13 (XPG_1), PF01984.15 (dsDNA binding domain), and PF01351.13 (RNase_H II).

**Gerd(archaeote) AC18** has 11 contigs, ranging from the longest 876,162 bp and the shortest 39,081. It has 1 archaea marker absent **- PF13685.8**/Fe-ADH_2, which was also absent in Lokiarchaeotes, and 7 duplications (PF03950.13/Glu/Gln-tRNA-synth_Ib, PF00749.16/ Glu/Gln-tRNA-synth_Ic, TIGR00134/GatD, TIGR2153/GatE, PF01351.13/RNase_HII, PF01981.11/PTH2, and TIGR00392/IleS).

**Odinarchaeote LCB_4**[5] was previously reported and has 9 contigs, ranging from the longest 1181447 bp to the shortest 5,866 bp. It has a duplication of the archaeal marker PF01984.15 (dsDNA binding domain), as in Thorarchaeotes.

## References


1 Paduan, J. B. *et al.* Discovery of Hydrothermal Vent Fields on Alarcón Rise and in Southern Pescadero Basin, Gulf of California. *Geochemistry, Geophysics, Geosystems* **19**, 4788-4819, doi:https://doi.org/10.1029/2018GC007771 (2018).

2 Imachi, H. *et al.* Isolation of an archaeon at the prokaryote–eukaryote interface. *Nature* **577**, 519-525, doi:10.1038/s41586-019-1916-6 (2020).

3 Caceres, E. F. *et al.* Near-complete Lokiarchaeota genomes from complex environmental samples using long and short read metagenomic analyses. *bioRxiv*, 2019.2012.2017.879148, doi:10.1101/2019.12.17.879148 (2019).

4 Manoharan, L. *et al.* Metagenomes from Coastal Marine Sediments Give Insights into the Ecological Role and Cellular Features of *Loki*- and *Thorarchaeota*. *mBio* **10**, e02039-02019, doi:10.1128/mBio.02039-19 (2019).

5 Zaremba-Niedzwiedzka, K. *et al.* Asgard archaea illuminate the origin of eukaryotic cellular complexity. *Nature* **541**, 353-358, doi:10.1038/nature21031 (2017).
