# Supplementary material for: Unique mobile elements and scalable gene flow at the prokaryote–eukaryote boundary revealed by circularized Asgard archaea genomes
Source: Nat Microbiol. 2022 Jan 13;7(2):200–12. doi: 10.1038/s41564-021-01039-y (PMC8813620; doi:10.1038/s41564-021-01039-y)
Supplement: Supplementary file 2 — Reporting Summary [file 41564_2021_1039_MOESM2_ESM.pdf]

nature research

Corresponding author(s): Fabai Wu & Victoria Orphan

Last updated by author(s): November 7, 2021

Reporting Summary

Nature Research wishes to improve the reproducibility of the work that we publish. This form provides structure for consistency and transparency in reporting. For further information on Nature Research policies, see our Editorial Policies and the Editorial Policy Checklist.

Statistics

For all statistical analyses, confirm that the following items are present in the figure legend, table legend, main text, or Methods section.

n/a	Confirmed	
☐	☒	The exact sample size (*n*) for each experimental group/condition, given as a discrete number and unit of measurement
☒	☐	A statement on whether measurements were taken from distinct samples or whether the same sample was measured repeatedly
☒	☐	The statistical test(s) used AND whether they are one- or two-sided<br>*Only common tests should be described solely by name; describe more complex techniques in the Methods section.*
☒	☐	A description of all covariates tested
☒	☐	A description of any assumptions or corrections, such as tests of normality and adjustment for multiple comparisons
☐	☒	A full description of the statistical parameters including central tendency (e.g. means) or other basic estimates (e.g. regression coefficient) AND variation (e.g. standard deviation) or associated estimates of uncertainty (e.g. confidence intervals)
☒	☐	For null hypothesis testing, the test statistic (e.g. *F*, *t*, *r*) with confidence intervals, effect sizes, degrees of freedom and *P* value noted<br>*Give P values as exact values whenever suitable.*
☒	☐	For Bayesian analysis, information on the choice of priors and Markov chain Monte Carlo settings
☒	☐	For hierarchical and complex designs, identification of the appropriate level for tests and full reporting of outcomes
☒	☐	Estimates of effect sizes (e.g. Cohen's *d*, Pearson's *r*), indicating how they were calculated

Our web collection on statistics for biologists contains articles on many of the points above.

Software and code

Policy information about availability of computer code

Data collection	Zen black version ELYRA was used for the acquisition of fluorescent images on Zeiss microscope.
Data analysis	DADA2 v1.9.1 ;R package(v3.6.0); canu v2.1; BamM v2.5.0; pilon v1.22; bedtools v2.29.2; LRScaf v1.1.10; SPAdes v3.14.1; metabat2 v2.15; MIRA v4 package; ANIcalculator v1.0; SINA v1.2.11; EggNOG mapper v2; cctyper 1.1.4; PSI-BLAST (https://blast.ncbi.nlm.nih.gov); CDD search (https://blast.ncbi.nlm.nih.gov); PHANNs (https://edwards.sdsu.edu/phanns); CheckM v1.1.3; hmmer v3.3.2; IQtree v2.1.2; UFBoot v2; MUSCLE v3.8.1551, anvi'o v6.2; ASM-Clust v1; blast v2.2.26; MAFFT v7.475; trimAl v1.4.1; minimap2 v2.17; catfasta2phyml (https://github.com/nylander/catfasta2phyml); custom script for amino acid recoding (https://github.com/dspeth/bioinfo_scripts/tree/master/phylogeny); custom matlab scripts under https://github.com/wufabai/genomics.

For manuscripts utilizing custom algorithms or software that are central to the research but not yet described in published literature, software must be made available to editors and reviewers. We strongly encourage code deposition in a community repository (e.g. GitHub). See the Nature Research guidelines for submitting code & software for further information.

Data

Policy information about availability of data

All manuscripts must include a data availability statement. This statement should provide the following information, where applicable:
- Accession codes, unique identifiers, or web links for publicly available datasets
- A list of figures that have associated raw data
- A description of any restrictions on data availability

The assembled genomes and raw metagenomic sequencing reads can be found on NCBI database under BioProject PRJNA721962, which was made publicly available on November 8, 2021.

Field-specific reporting

Please select the one below that is the best fit for your research. If you are not sure, read the appropriate sections before making your selection.

☐ Life sciences ☐ Behavioural & social sciences ☒ Ecological, evolutionary & environmental sciences

For a reference copy of the document with all sections, see nature.com/documents/nr-reporting-summary-flat.pdf

Ecological, evolutionary & environmental sciences study design

All studies must disclose on these points even when the disclosure is negative.

Study description	Anaerobic laboratory cultivation using artificial sea water
Research sample	Sediment and Rocks collected from hydrothermal vents. The samples were chosen due to their geographical proximity to the vents with diffusive venting, which provide nutrients that fuel the local ecosystem.
Sampling strategy	Samples were collected in an anaerobic chamber using pipettes. Sample sizes were empirical determined, typically 1ml in volume, to allow extraction of sufficient amount of DNA while causing the least amount of disturbance to the existing microbiome.
Data collection	16S rRNA Amplicon sequencing data using Illumina MiSeq were collected by Laragen. Full-Length 16S rRNA Sequencing data using PacBio Sequel II were collected by Brigham Young University Sequencing Center. Metagenomic sequencing data via Illumina HiSeq2000 were collected by Novogen. Metagenomic sequencing data via Oxford Nanopore MinION were collected by author Igor A. Antoshechkin.
Timing and spatial scale	The sampling of the initial rock and sediment samples were respectively carried out at the Auka vent field, Pescadero basin, Mexico on November 2, 2017 and on November 14, 2018. The sampling of rock incubations were sampled inside of the anaerobic chamber at Caltech between November 8, 2018 and December 15, 2019 with an increasing interval from 3 weeks to 8 months. The exact dates are specified in Supplementary Table 2. The sediment incubations were sampled on date June 23, July 29, and September 23, 2019.
Data exclusions	All sequencing data were used for analyses without exclusion.
Reproducibility	The paper focuses on bioinformatics analyses, and all analyses can be reproduced using publicly available software packages provided in the Methods section. The DNA samples were analyzed twice during the rock incubation at 2-4 months around the time when the AAG phylotypes started to emerge. No specific incubation conditions had experimental replicates.
Randomization	The experiments were designed to discover novel organisms from any possible condition. The work does not focus on the effect of environmental parameters.
Blinding	We do not carry out randomized testing on experimental subjects, as the experiments were designed to discover novel organisms from any possible condition. There is no visual link between the samples and the microbes of interest, and there is a minimum of 2 months between the time of sampling and the time of sequencing data output, blinding neither increase nor decrease bias.

Did the study involve field work? ☒ Yes ☐ No

Field work, collection and transport

Field conditions	Field sites are 3.6 km below sea level, collected at natural conditions on the dates and location provided in the Methods section. The local temperature were measure at around 40 °C, although with uncertainty due to the strong temperature gradient at the sampling site.
Location	[23°57'N; 108°51'W] [23°57'N; 108°52'W] [23°53'N; 108°48'W]
Access & import/export	Sample collection accompanied by and under the construction local scientists under permission granted by local government. Sample collection permits for the expedition was granted by la Dirección General de Ordenamiento Pesquero y Acuícola, Comisión Nacional de Acuacultura y Pesca (CONAPESCA: Permiso de Pesca de Fomento No. PPFE/DGOPA-200/18) and la Dirección General de Geografía y Medio Ambiente, Instituto Nacional de Estadística y Geografía (INEGI: Autorización EG0122018), with the associated Diplomatic Note number 18-2083 (CTC/07345/18) from la Secretaría de Relaciones Exteriores - Agencia Mexicana de Cooperación Internacional para el Desarrollo / Dirección General de Cooperación Técnica y Científica. The permit EG0072017 for the 2017 cruise was granted on April 18, 2017. The permit EG0122018 for the 2018 cruise was granted on July 25, 2018.
Disturbance	Samples were collected outside the major chimney area to result in minimal influence on the macrofauna and the structural integrity of the chimneys.

Reporting for specific materials, systems and methods

We require information from authors about some types of materials, experimental systems and methods used in many studies. Here, indicate whether each material, system or method listed is relevant to your study. If you are not sure if a list item applies to your research, read the appropriate section before selecting a response.

Materials & experimental systems

n/a	Involved in the study
☒	☐ Antibodies
☒	☐ Eukaryotic cell lines
☒	☐ Palaeontology and archaeology
☒	☐ Animals and other organisms
☒	☐ Human research participants
☒	☐ Clinical data
☒	☐ Dual use research of concern

Methods

n/a	Involved in the study
☒	☐ ChIP-seq
☒	☐ Flow cytometry
☒	☐ MRI-based neuroimaging
