# Supplementary material for: Unique mobile elements and scalable gene flow at the prokaryote–eukaryote boundary revealed by circularized Asgard archaea genomes
Source: Nat Microbiol. 2022 Jan 13;7(2):200–12. doi: 10.1038/s41564-021-01039-y (PMC8813620; doi:10.1038/s41564-021-01039-y)
Supplement: Supplementary file 3 — Peer Review Information [file 41564_2021_1039_MOESM3_ESM.pdf]

natureresearch

Peer Review Information

Journal: Nature Microbiology
Manuscript Title: Unique mobile elements and scalable gene flow at the prokaryote-eukaryote boundary revealed by circularized Asgard archaea genomes
Corresponding author name(s): Fabai Wu

Reviewer Comments & Decisions:

Decision Letter, initial version:

Dear Victoria, dear Fabai,

Thank you for your patience while your manuscript "Evolutionary Plasticity of Archaea at the Eukaryotic Root" was under peer-review at Nature Microbiology. It has now been seen by 3 referees, whose expertise and comments you will find at the end of this email. As you will see from their comments, although they find your work of potential interest, they have raised a number of concerns that will need to be addressed before we can consider publication of the work in Nature Microbiology.

In particular, you will see that while all referees praise the enrichments, new genomes and the analyses based on these data, they also make multiple suggestions for improvement, particularly in terms of streamlining the main messages and performing some additional analyses to further substantiate some of the main claims. For example, referee #1 suggests revising the placement using a more comprehensive set of publicly available genomes (or otherwise tone down the current claims accordingly); referee #2 suggests testing more formally whether the core archaeal genes are playing essential conserved roles in Asgards, whereas gene acquisitions from bacteria and ESPs are more niche-relevant or generally less conserved; referee #2 also says it may be useful to quantify the support for an Asgard origin of these genes in eukaryotes using approximately-unbiased tests, or another appropriate tree selection test; and referee #3 suggests additional analyses to support the claim that the mobilome is involved in eukaryogenesis through passage of genes from bacteria to the nascent eukaryotic cell. As you will see, referee #3 also suggests some microscopy-based assessment of the enrichments, particularly in light of past work documenting protrusions in Promethearchaeum, yet we would like to clarify that although we agree that such analyses may be interesting and informative, they would not be required for subsequent consideration of your manuscript for publication in Nature Microbiology.

In addition to these requests for additional analyses, the referees also make several suggestions on

how to streamline the paper - these seem quite clear and straight forward to address. Should further experimental data and text modifications allow you to address these criticisms, we would be very happy to look at a revised manuscript. We are committed to providing a fair and constructive peer-review process, so please do not hesitate to contact us if there are specific requests from the reviewers that you believe are technically impossible or unlikely to yield a meaningful outcome.

We strongly support public availability of data. Please place the data used in your paper into a public data repository, if one exists, or alternatively, present the data as Source Data or Supplementary Information. If data can only be shared on request, please explain why in your Data Availability Statement, and also in the correspondence with your editor. For some data types, deposition in a public repository is mandatory - more information on our data deposition policies and available repositories can be found at https://www.nature.com/nature-research/editorial-policies/reporting-standards#availability-of-data.

Please include a data availability statement as a separate section after Methods but before references, under the heading "Data Availability". This section should inform readers about the availability of the data used to support the conclusions of your study. This information includes accession codes to public repositories (data banks for protein, DNA or RNA sequences, microarray, proteomics data etc…), references to source data published alongside the paper, unique identifiers such as URLs to data repository entries, or data set DOIs, and any other statement about data availability. At a minimum, you should include the following statement: "The data that support the findings of this study are available from the corresponding author upon request", mentioning any restrictions on availability. If DOIs are provided, we also strongly encourage including these in the Reference list (authors, title, publisher (repository name), identifier, year). For more guidance on how to write this section please see:
http://www.nature.com/authors/policies/data/data-availability-statements-data-citations.pdf

When revising your manuscript:

* Include a "Response to referees" document detailing, point-by-point, how you addressed each referee comment. If no action was taken to address a point, you must provide a compelling argument. This response will be sent back to the referees along with the revised manuscript.

* If you have not done so already we suggest that you begin to revise your manuscript so that it conforms to our Article format instructions at http://www.nature.com/nmicrobiol/info/final-submission. Refer also to any guidelines provided in this letter.

* Include a revised version of any required reporting checklist. It will be available to referees (and, potentially, statisticians) to aid in their evaluation if the manuscript goes back for peer review. A revised checklist is essential for re-review of the paper.

When submitting the revised version of your manuscript, please pay close attention to our href="https://www.nature.com/nature-research/editorial-policies/image-integrity">Digital Image Integrity Guidelines. and to the following points below:

-- that unprocessed scans are clearly labelled and match the gels and western blots presented in figures.

-- that control panels for gels and western blots are appropriately described as loading on sample processing controls
-- all images in the paper are checked for duplication of panels and for splicing of gel lanes.

Finally, please ensure that you retain unprocessed data and metadata files after publication, ideally archiving data in perpetuity, as these may be requested during the peer review and production process or after publication if any issues arise.

Please use the link below to submit a revised paper:

{redacted}

Note: This url links to your confidential homepage and associated information about manuscripts you may have submitted or be reviewing for us. If you wish to forward this e-mail to co-authors, please delete this link to your homepage first.

Nature Microbiology is committed to improving transparency in authorship. As part of our efforts in this direction, we are now requesting that all authors identified as 'corresponding author' on published papers create and link their Open Researcher and Contributor Identifier (ORCID) with their account on the Manuscript Tracking System (MTS), prior to acceptance. This applies to primary research papers only. ORCID helps the scientific community achieve unambiguous attribution of all scholarly contributions. You can create and link your ORCID from the home page of the MTS by clicking on 'Modify my Springer Nature account'. For more information please visit please visit www.springernature.com/orcid.

If you wish to submit a suitably revised manuscript we would hope to receive it within 6 months. If you cannot send it within this time, please let us know. We will be happy to consider your revision, even if a similar study has been accepted for publication at Nature Microbiology or published elsewhere (up to a maximum of 6 months).

In the meantime we hope that you find our referees' comments helpful.

With best regards,

{redacted}

****
Reviewer Comments:

Reviewer #1 (Remarks to the Author):

Overall, this study is a very interesting read and innovative, and the reconstruction of two complete Heimdallarchaeota genomes is an important contribution to our understanding of the Asgard archaea. I do believe it deserves to be in Nature Micro, however there are some fairly issues that need to be resolved:

The authors state that these complete genomes are "phylogenetically closest to eukaryotes", however their phylogenies only include very few of the dozens of genomes available. It has been suggested

that Heimdall are closest to eukaryotes in various other publications, but this is not well resolve yet. It is an open question, and a very important one! Their phylogeny supports this; however, it doesn't include a comprehensive set now publicly available, there may be other lineages not included that may be more closely associated with the eukaryote branch. I know first-hand this is not a small undertaking (to resolve the position of eukaryotes) with many genomes. So, I would back off on these statements, or run additional phylogenies with more MAGs.

I have concerns about the identity of some of the CRISPR/Cas-containing contigs (shown in figure 2 and in text ln 203). MAG B51_G16 (GCA_003650225.1) is a Chloroflexi bacteria and Figure 2f B53_G16 (GCA_003660845.1) is a Pacearchaeota. Both are from NCBI PRJNA362212 and associated Guaymas Basin publications. Both of these identities are supported by Dombrowski et al. 2018 and GTDB. Perhaps these contigs were miss-binned in the original publication, or is there co-infection occurring? This is something that should be investigated and explained more.

Sample location for homologs of AagV1-7. In Figure 2a (E44_bin34 or GCA_004376455.1) "homologs of the AagV1-7 protein" and in text, pg. 9 and reference 13 is for the Eastern Gulf of Mexico, Atlantic Ocean (26.28 N 86.81 W)

****
Reviewer #2 (Remarks to the Author):

In this manuscript, Wu et al. sequence, assemble and analyse 8 new Asgard archaea genomes, and perform comparative analyses that bear on archaeal genome evolution and eukaryogenesis.

The most significant contributions of the manuscript, all of which are in my view of major importance, are:
(i) the generation, through culture enrichment, of two new complete Heimdallarchaeota genomes, but the six other high-quality new genomes are also very welcome and useful data for understanding Asgard biology and studying questions around eukaryogenesis;
(ii) comparative genomic analyses of these high-quality genomes that confirm previous findings that Asgards encode various "eukaryote-specific" (or eukaryote-related) proteins, and may have donated these to eukaryotes via the Asgard-derived host cell for the mitochondrial endosymbiont (Note: though in some respects confirmatory, this kind of confirmation is actually very important for the field at the moment, given ongoing debates about the quality of the published Asgard MAGs).
(iii) The identification and bioinformatic analysis of virus-like integrated elements with intriguing biology in Heimdallarchaeota, and more generally an analysis of the "mobilome" of these Asgards, which is now possible thanks to the high quality of these assemblies relative to published MAGs.

This is an excellent and timely analysis that will make a real difference in ongoing discussions about eukaryogenesis, but which also provides interesting new data on the genome biology of Asgards. In general, the methods are appropriate (caveat: those that I am qualified to judge, particularly the phylogenetics and comparative genomics) and the interpretations are well-founded. Some more detailed/specific comments follow.

1. Lines 289-90: The eukaryote root and the placement of the eukaryote nuclear lineage within the Asgard archaea are distinct concepts (with the eukaryote root usually used to refer to ideas about the deepest split within eukaryotes, not their relation to archaea or bacteria). I suggest reformulating here

to avoid confusion, e.g. "This is in agreement with recent analyses placing the eukaryotic nuclear lineage/host cell for the mitochondrial endosymbiont within the Asgard archaea" or "This is in agreement with recent analyses supporting a close specific relationship between eukaryotes and Heimdallarchaeota", or similar.For the same reason, it may be worth re-considering the title of the manuscript to make clear that it is eukaryogenesis/the relationship of eukaryotes and Archaea, and not the inter-relationships among eukaryotic groups, that is being addressed

2. Lines 315-322: The parallel drawn between the finding of ESPs and especially bacteria-origin genes acting as a kind of accessory "cloud" of genes and the situation in eukaryotes is interesting, but needs to be unpacked and set out with greater specificity and detail. In eukaryotes, the finding that species with fewer genes tend to be enriched for archaeal-origin genes has been explained either by the increased functional importance of archaeal-origin genes (e.g. as informational genes), or alternatively due to loss of the mitochondrion (and so a large tranche of bacterial-origin genes) in the most reduced eukaryotic lineages such as Microsporidia. Presumably, the hypothetical mechanistic basis here is the former: that the core archaeal genes are playing essential conserved roles in Asgards, whereas gene acquisitions from bacteria and ESPs are more niche-relevant or generally less conserved. If so, this should be stated clearly and, if possible, tested against the data presented in Figure 4. Do the functions of the 900 core archaeal genes differ from those of the bacteria-origin genes? How does the situation compare to the "half-lives" of horizontally-acquired genes more generally, which are often lost soon after they are acquired (e.g. van Passel et al. 2008 https://journals.plos.org/ploscompbiol/article?id=10.1371/journal.pcbi.1000059)

3. In Figure 4(c), I found the schematic of the new hypothesis unclear. The "ancestral Asgard" presumably existed before the first Heimdall archaea, yet the ancestor is shown as donating genes along the FECA to LECA stem. Perhaps this would be clearer if the donor were labelled as "related Asgard archaea" or similar (if this is the intended meaning...).

4. One of the exciting proposals in the study is that some of the bacterial-origin genes in eukaryotes might have been acquired via the archaeal host, or one of its relatives (i.e., an "indirect" acquisition from bacteria). However, the gene trees provided in Extended Figure 10 in support of these individual cases are, with the exception of (d), perhaps not very compelling. This is not surprising given the difficulty of inferring robust single gene trees, particularly for anciently diverged sequences, but these results should perhaps be discussed more cautiously in the main text. It might be useful to quantify the support for an Asgard origin of these genes in eukaryotes using approximately-unbiased tests, or another appropriate tree selection test. The likelihood of the ML tree in which the Asgard+eukaryote sequences (or a subset of them) form a clade to the exclusion of Bacteria could be compared to the ML tree in which the closest bacterial group forms a clade with the eukaryotes.

Minor points:

1. Define "mobilome" in the abstract when first used, or reformulate to avoid this perhaps field-specific term.
2. In Extended Figure 8, I think the author of the 2013 study was "Alvarez-Ponce".

****
Reviewer #3 (Remarks to the Author):

The paper by Wu and colleagues reports further interesting genomic insights into the Asgard archaea.

They enriched two members of Heimdallarchaeota, currently the clade that is closest to eukaryotes and named them Ca. Heimdallaerchaeum endolithica and Ca Heimdallarchaeum aukensis. This allowed them to obtain complete circularized genomes from these two strains.

This is a very important result as isolation of Asgard representatives is one of the highest priorities in the archaeal field. Presently, as far as I understand, only one enriched and sequenced member of Asgard in available (Promethearchaeum).

The paper is therefore surely an important one, but my general impression is that it lacks focus, and does not really know which way to go. Even the abstract does not really reflect the content, as a large part of the results is focused on proviruses and mobile elements. I commend the authors for wanting to make a new scenario for the emergence of eukaryotes, but I fear that this does not rely strongly on the analyses and is not discussed in detail. In this respect, the very title of the paper does not reflect the content of the article.
The main message to me is that the authors obtained two enrichments and they analyzed in detail the mobilome, and this is original enough that the paper could be clearly built around it. The rest of results are presented as confirmations of previous data (placement with respect to, eukaryotes, presence of ESPs). Moreover, if the authors want to claim that this mobilome is involved in eukaryogenesis through passage of genes from bacteria to the nascent eukaryotic cell, this must be supported much more by the data and discussed in depth, clearly showing that these mobilomes do vehicle genes from bacteria to eukaryotes (e.g., that some of these genes are embedded in mobile elements? by one clear phylogeny as main figure?). This could be an extremely powerful message.
Also, I was disappointed that the authors have two beautiful enrichments in their hands but do not attempt at more detailed microscopy data. This could be extremely interesting to see for example, if the same protrusions are observed as in Promethe.
I might be wrong, but I think the authors are not citing nor comparing their results to those of the recent Nature paper by Koonin and Meng Li. This is important.
Finally, I found the figures complex (figure 2 in particular) and not very well explained in the text.

Below a few comments to help improve readability and impact.

This is a personal impression, but I feel that the text is structured somehow erratically.

I think the first thing the readers want to see is a tree where these two genomes are placed, and this instead comes very late in Figure 3.
Then I would move on to describe what is new in terms of ESPs in these genomes (basically move here the paragraph from lines 265-303), and then move on to the most original results (the mobilome and the gene flux), which would nicely lead to the proposed hypothesis (although substantiated by more analysis).

Also, the first paragraph is very hard to follow in the absence of a main figure.

Line 102, this could be a new paragraph presenting a description of the genome contents. Here, there is a sentence that could be completed by some discussion: line 116, what is the missing enzyme for ester-linked lipids for? what does its absence imply? It is also absent from other Asgard genomes? The authors mention a mosaic distribution of these enzymes but do not really discuss it.

Line 132: why is this intriguing?

Line 137: did you try to reassemble by using your method?

Line 143: say more clearly that they are from bacteria and mention which bacteria.

A large part of the results presents an extensive analysis of integrated proviruses in the two circular genomes. This is very interesting and as I said, should be highlighted more in both the title and the abstract. Lines 250-256, please include a main figure and more detailed results to support your claims at lines 256-258).

Line 305, the analysis of gene flux scaling is nice and new, and could certainly be further developed. The possibility that the genome sized of the other MAGs are affected by assembly problems is not evoked.

Line 330: interesting model, which could be merged with the last paragraph as a common discussion. However, as I said earlier, I do not think it is strongly supported by the data, or at least not as presented in the current text.

Please include a full etymology of the two candidatus at the end of the paper, so to protect yourself against a recent tendency by some people to change prokaryotic taxonomy (and names), creating a lot of confusion.

Finally, be extra careful to provide all raw metagenomic data to the readership.

Author Rebuttal to Initial comments

Reviewer #1 (Remarks to the Author):

Overall, this study is a very interesting read and innovative, and the reconstruction of two complete Heimdallarchaeota genomes is an important contribution to our understanding of the Asgard archaea. I do believe it deserves to be in Nature Micro,

We thank the review for the positive comments on our manuscript and finding it an important contribution to the field. According to reviewers' comments, we have restructured and streamlined the paper to improve focus and clarity to support the main conclusions of the paper.

however there are some fairly issues that need to be resolved:

The authors state that these complete genomes are "phylogenetically closest to eukaryotes", however their phylogenies only include very few of the dozens of genomes available. It has

been suggested that Heimdall are closest to eukaryotes in various other publications, but this is not well resolve yet. It is an open question, and a very important one! Their phylogeny supports this; however, it doesn't include a comprehensive set now publicly available, there may be other lineages not included that may be more closely associated with the eukaryote branch. I know first-hand this is not a small undertaking (to resolve the position of eukaryotes) with many genomes. So, I would back off on these statements, or run additional phylogenies with more MAGs.

We acknowledge the complexity in the phylogenetic placement of eukaryotes, and it is an ongoing effort. After consulting experts who are currently working on this problem, we have now both sampled a larger range of Asgard archaea lineages to support our conclusion and tempered claims of relatedness with eukaryotes, now better acknowledging that it is still an ongoing effort.

First, we explained in more detail the existing taxonomy within the Heimdall group archaea that have influenced the scope of Heimdallarchaeota, including the drastic differences between the findings from the recent publications of Liu et al Nature 2021 and Rinke et al Nature Microbiology 2021. We performed phylogenomic analyses using all publicly available Heimdall-related genomes (as of August 2021) and demonstrated that our circular genomes cluster within the Heimdall clade. Our updated analysis is now included in Fig. 1b along with companion text in (83-92).

To expand our taxonomic sampling, we conducted new analyses with a total of 282 asgard archaeal genomes available from public databases, including the most recent Liu et al Nature 2021 and Sun et al ISME Comm 2021, which substantially expanded Asgard diversity. We constructed a phylogenomic tree of all Asgard archaeal genomes using TACK as outgroup (Fig. S2). We also constructed new HMMs that performed much better than existing HMMs available in the CheckM package, which sporadically misses gene markers due to the divergence of Asgard proteins from the Euryarchaeota and Crenarchaeota that were used to construct the old HMMs. This increased the overall estimates of completeness, as well as redundancy scores for the Asgard genomes, which we provide as supplementary table 8 and 9. This is now described in lines 136-139 and in the Methods section. The HMMs are also provided as supplementary resources on Figshare.

Next, we applied a less stringent criterion to sample across Asgard lineages with evenness in mind. The final selected genomes for this analysis are highlighted in supplementary table 9. In addition to the original tree shown as the main panel in Fig. 1d, we now constructed a new

phylogenomic tree based on these selected genomes. This analysis covered majority of Asgard lineages, although there were a few lineages, Kai, Wukong, and Jord, lacking high quality genomes that did not pass our filtering criteria. For example, Wukongarchaeota from Liu et al 2011 Nature has 3 assemblies of the same species, which all contain nearly 300 contigs. However, we do not think that it will influence our general claim. Our new tree (Fig. S4) supports the close relationship between the Heimdall group and eukaryotes. Updated text can be found in lines 139-142.

Based on these new analyses, we conclude that our phylogeny supports previously reported placement of Heimdall group as a close relative to eukaryotes, while also acknowledging that the statistical approach and taxonomic sampling is likely to continue to improve with additional environmental sequencing of archaea and eukaryotic crown groups in the future. We highlight the value of constructing and evaluating high quality genomes as provided in our study, and hope that it will play a positive role in future efforts in phylogenomics. Additions to the text can be found in lines 142-146.

I have concerns about the identity of some of the CRISPR/Cas-containing contigs (shown in figure 2 and in text ln 203). MAG B51_G16 (GCA_003650225.1) is a Chloroflexi bacteria and Figure 2f B53_G16 (GCA_003660845.1) is a Pacearchaeota. Both are from NCBI PRJNA362212 and associated Guaymas Basin publications. Both of these identities are supported by Dombrowski et al. 2018 and GTDB. Perhaps these contigs were miss-binned in the original publication, or is there co-infection occurring? This is something that should be investigated and explained more.

We thank the reviewer for pointing out this discrepancy. The B51_G16 was indeed a typo and we meant it as B53_G16, which was shown in the figure. We recognize that B53_G16 from Guaymas Basin is a highly fragmented and incomplete genome, but we found it encodes nearly identical proteins with our Ca. H. endolithica from Pescadero Basin. We thus performed ANI analyses and found that in fact it was so close to the latter that they should be classified as the same species. We now clarified early on in the text (lines 91-94) and re-classified this MAG. We additionally commented on the fact that this genome was recovered from Guaymas basin, a hydrothermal vent system ~400 km from Pescadero basin in the Gulf of California (line 223-228).

Sample location for homologs of AagV1-7. In Figure 2a (E44_bin34 or GCA_004376455.1) “homologs of the AagV1-7 protein” and in text, pg. 9 and reference 13 is for the Eastern Gulf of Mexico, Atlantic Ocean (26.28 N 86.81 W)

We have now revised the text (line 228-231) and within the new figure panel Fig. 3e to indicate the origin in the Gulf of Mexico.

****

Reviewer #2 (Remarks to the Author):

In this manuscript, Wu et al. sequence, assemble and analyse 8 new Asgard archaea genomes, and perform comparative analyses that bear on archaeal genome evolution and eukaryogenesis.

The most significant contributions of the manuscript, all of which are in my view of major importance, are:
(i) the generation, through culture enrichment, of two new complete Heimdallarchaeota genomes, but the six other high-quality new genomes are also very welcome and useful data for understanding Asgard biology and studying questions around eukaryogenesis;
(ii) comparative genomic analyses of these high-quality genomes that confirm previous findings that Asgards encode various "eukaryote-specific" (or eukaryote-related) proteins, and may have donated these to eukaryotes via the Asgard-derived host cell for the mitochondrial endosymbiont (Note: though in some respects confirmatory, this kind of confirmation is actually very important for the field at the moment, given ongoing debates about the quality of the published Asgard MAGs).
(iii) The identification and bioinformatic analysis of virus-like integrated elements with intriguing biology in Heimdallarchaeota, and more generally an analysis of the "mobilome" of these Asgards, which is now possible thanks to the high quality of these assemblies relative to published MAGs.

This is an excellent and timely analysis that will make a real difference in ongoing discussions about eukaryogenesis, but which also provides interesting new data on the genome biology of Asgards. In general, the methods are appropriate (caveat: those that I am qualified to judge, particularly the phylogenetics and comparative genomics) and the interpretations are well-founded. Some more detailed/specific comments follow.

We thank the reviewer for the positive comments on the manuscript. According to reviewers' comments, we have restructured and streamlined the paper to improve focus and clarity to support the main conclusions of the paper.

1. Lines 289-90: The eukaryote root and the placement of the eukaryote nuclear lineage within the Asgard archaea are distinct concepts (with the eukaryote root usually used to refer to ideas about the deepest split within eukaryotes, not their relation to archaea or bacteria). I suggest reformulating here to avoid confusion, e.g. "This is in agreement with recent analyses placing

the eukaryotic nuclear lineage/host cell for the mitochondrial endosymbiont within the Asgard archaea" or "This is in agreement with recent analyses supporting a close specific relationship between eukaryotes and Heimdallarchaeota", or similar. For the same reason, it may be worth re-considering the title of the manuscript to make clear that it is eukaryogenesis/the relationship of eukaryotes and Archaea, and not the inter-relationships among eukaryotic groups, that is being addressed

We thank the reviewer for pointing this out and we agree that the use of ‘eukaryotic root’ is confusing. The revised manuscript avoids use of this term in the title, abstract, and the main text, now replaced with more specific descriptions as suggested throughout.

2. Lines 315-322: The parallel drawn between the finding of ESPs and especially bacteria-origin genes acting as a kind of accessory "cloud" of genes and the situation in eukaryotes is interesting, but needs to be unpacked and set out with greater specificity and detail. In eukaryotes, the finding that species with fewer genes tend to be enriched for archaeal-origin genes has been explained either by the increased functional importance of archaeal-origin genes (e.g. as informational genes), or alternatively due to loss of the mitochondrion (and so a large tranche of bacterial-origin genes) in the most reduced eukaryotic lineages such as Microsporidia. Presumably, the hypothetical mechanistic basis here is the former: that the core archaeal genes are playing essential conserved roles in Asgards, whereas gene acquisitions from bacteria and ESPs are more niche-relevant or generally less conserved. If so, this should be stated clearly and, if possible, tested against the data presented in Figure 4. Do the functions of the 900 core archaeal genes differ from those of the bacteria-origin genes? How does the situation compare to the "half-lives" of horizontally-acquired genes more generally, which are often lost soon after they are acquired (e.g. van Passel et al. 2008 https://journals.plos.org/ploscompbiol/article?id=10.1371/journal.pcbi.1000059)

We thank the reviewer for the fantastic suggestion. We have now performed functional analyses on the three different taxonomic groups, and as shown in our newly reconfigured Fig. 5a, indeed the three groups have different functional distributions. These results are now detailed in (lines 270-283). Regarding whether the comparisons to the ‘half-lives’ of horizontally transferred genes, we find it technically challenging at present, as different from the example provided in van Passel et al, where many E. coli strains were compared with each other, we currently only have 2 closely related Heimdall species here. The strain-level diversity in Asgard archaea is absolutely an interesting aspect to study in the future, and likely will provide further statistical support for our notion of pan-Asgard ERPs. We have discussed the issue of timescale in the Discussion section (line 346-350)

3. In Figure 4(c), I found the schematic of the new hypothesis unclear. The "ancestral Asgard"

presumably existed before the first Heimdall archaea, yet the ancestor is shown as donating genes along the FECA to LECA stem. Perhaps this would be clearer if the donor were labelled as "related Asgard archaea" or similar (if this is the intended meaning...).

We have replaced ‘ancestral Asgard’ in the figure by ‘related Asgard archaea’, which is now in panel Fig. 6c.

4. One of the exciting proposals in the study is that some of the bacterial-origin genes in eukaryotes might have been acquired via the archaeal host, or one of its relatives (i.e., an "indirect" acquisition from bacteria). However, the gene trees provided in Extended Figure 10 in support of these individual cases are, with the exception of (d), perhaps not very compelling. This is not surprising given the difficulty of inferring robust single gene trees, particularly for anciently diverged sequences, but these results should perhaps be discussed more cautiously in the main text. It might be useful to quantify the support for an Asgard origin of these genes in eukaryotes using approximately-unbiased tests, or another appropriate tree selection test. The likelihood of the ML tree in which the Asgard+eukaryote sequences (or a subset of them) form a clade to the exclusion of Bacteria could be compared to the ML tree in which the closest bacterial group forms a clade with the eukaryotes.

We appreciate the helpful and detailed advice from the reviewer for improving the statistical support of the proposed ‘indirect acquisition’ scenario. While the statistical support is weak in some cases, if we look at the detailed placement in the phylogenetic tree (which we now find can vary depending on the model used), the evolutionary trajectory is quite fascinating, as Asgard archaea are often the only Archaea taxa that stand between bacteria and eukaryotes.

However, given the new analyses and text supporting other findings in our manuscript, we realized there is no room in this manuscript to sufficiently unpack the logic in the text or highlight this hypothesis in the abstract. We thus decided to remove this component in our proposal of eukaryotic origin in the current revision. We have mentioned it as an interesting direction in the Discussion section (line 356-359). In the future, we hope to expand these analyses to include all archaea which will offer a more comprehensive study and hopefully a more robust conclusion. We hope that the reviewer finds this decision a reasonable one.

Minor points:

1. Define "mobilome" in the abstract when first used, or reformulate to avoid this perhaps field-specific term.

We have replaced the term using ‘mobile elements’ throughout, except one mention in the discussion section, where we fully explain this term.

2. In Extended Figure 8, I think the author of the 2013 study was "Alvarez-Ponce".

Thank you. We have corrected the author’s name and now placed the SI figure panel in the main figure Fig. 5b.

****
Reviewer #3 (Remarks to the Author):

The paper by Wu and colleagues reports further interesting genomic insights into the Asgard archaea.

They enriched two members of Heimdallarchaeota, currently the clade that is closest to eukaryotes and named them Ca. Heimdallaerchaeum endolithica and Ca Heimdallarchaeum aukensis. This allowed them to obtain complete circularized genomes from these two strains.

This is a very important result as isolation of Asgard representatives is one of the highest priorities in the archaeal field. Presently, as far as I understand, only one enriched and sequenced member of Asgard in available (Promethearchaeum).

We thank the reviewer for the positive comment on the manuscript.

The paper is therefore surely an important one, but my general impression is that it lacks focus, and does not really know which way to go. Even the abstract does not really reflect the content, as a large part of the results is focused on proviruses and mobile elements. I commend the authors for wanting to make a new scenario for the emergence of eukaryotes, but I fear that this does not rely strongly on the analyses and is not discussed in detail. In this respect, the very title of the paper does not reflect the content of the article.

We thank the reviewer for providing critical comments and suggestions to help us improve the flow of the paper. We have now restructured the paper to improve clarity and focus as suggested by the reviewer in their comments below. We also substantially edited the abstract to reflect the central findings of the manuscript more closely.

The main message to me is that the authors obtained two enrichments and they analyzed in

detail the mobilome, and this is original enough that the paper could be clearly built around it. The rest of results are presented as confirmations of previous data (placement with respect to, eukaryotes, presence of ESPs). Moreover, if the authors want to claim that this mobilome is involved in eukaryogenesis through passage of genes from bacteria to the nascent eukaryotic cell, this must be supported much more by the data and discussed in depth, clearly showing that these mobilomes do vehicle genes from bacteria to eukaryotes (e.g., that some of these genes are embedded in mobile elements? by one clear phylogeny as main figure?). This could be an extremely powerful message.

We have now expanded the mobilome section to 3 figures (Fig. 2-4). Upon reanalysis using the most updated viral database IMGVR3 and genome database GTDB, we now report a clearer phylogenetic signal of viral genes, which relate to bacteriophages infecting Bacteriodota and Firmicutes (Fig. 4a-b) and Fig. S8. We now also provided examples of Heimdallarchaeal mobile elements garnering genes from bacteria as well as Asgard archaea hosts. Regarding the direct evidence of ESPs embedded within mobilomes, we currently do not have a clear example that can be highlighted. We believe this is because 1) we are only detecting a small fraction of the mobilome using our approach and the full spectrum of mobilome features for Asgard archaea are yet to be revealed, and 2) the genes that were passed from bacteria into the Asgard/eukaryotes at the onset of eukaryogenesis have been there for so long that they may no longer be part of the present-day mobile elements. They may, however, be moving around as part of their pangenome through other means such as conjugation and homologous recombination. The latter is in line with our analyses of ERPs in Fig. 6. We thus decided to not make the direct link between them in the present text and instead emphasize the importance of timescale in the Discussion (line 346-350).

Related to ESPs vs mobilome, we now revised the text to state that: *'They are likely, or could have been during their evolutionary history, shuffled as part of their mobilome elements.'* (lines 326) We now highlight two independent cases that fall under the umbrella of horizontal transfer: 1) Asgard archaea have bacterial input just like eukaryotes have bacterial input, and they both scale the same with genome size (Fig. 5b); 2) eukaryote-related genes in Asgards are only partially conserved and is genome size-dependent (Fig. 5c and Fig. 6a-b).

Also, I was disappointed that the authors have two beautiful enrichments in their hands but do not attempt at more detailed microscopy data. This could be extremely interesting to see for example, if the same protrusions are observed as in Promethe.

We agree that morphological descriptions of these archaea are of high importance and this was part of our initial efforts. We designed several FISH probes specific for our Heimdallarchaeota, but have not been able to confidently image the Heimdallarchaeum spp. in our enrichments unfortunately. We suspect that it may have been due to their physical association with the rock/sediments, which made them hard to separate for imaging. As a side note, we also recently attempted the use probe Heim1-526 described in Salcher et al mSphere 2020, but this probe appears to target a *Methanolobus* archaea in our microbial community. We will continue to work towards imaging these organisms and hope that further enrichment will allow us to acquire information about their morphology and ultrastructural organization.

I might be wrong, but I think the authors are not citing nor comparing their results to those of the recent Nature paper by Koonin and Meng Li. This is important.

We have cited their paper in the previous version of the manuscript for their finding of ESPs. In our revised manuscript, we have fully analyzed all genomes available from their study and referenced this work.

Finally, I found the figures complex (figure 2 in particular) and not very well explained in the text.

We have now expanded our manuscript from 4 figures to 6 figures and improved their descriptions in the captions and the text.

Below a few comments to help improve readability and impact.

This is a personal impression, but I feel that the text is structured somehow erratically.

I think the first thing the readers want to see is a tree where these two genomes are placed, and this instead comes very late in Figure 3.
Then I would move on to describe what is new in terms of ESPs in these genomes (basically move here the paragraph from lines 265-303), and then move on to the most original results (the mobilome and the gene flux), which would nicely lead to the proposed hypothesis (although substantiated by more analysis).

Also, the first paragraph is very hard to follow in the absence of a main figure.

We thank the reviewer for helping us streamlining the flow of the manuscript. We have now accordingly moved the entire section forward and composed a new Figure 1 that is more reflective of the main message of this section and serves to guide the readers. We have moved the original text in lines 265-303 forward. The new text associated with Figure 1 can now be found in lines 53-146.

Line 102, this could be a new paragraph presenting a description of the genome contents. Here, there is a sentence that could be completed by some discussion: line 116, what is the missing enzyme for ester-linked lipids for? what does its absence imply? It is also absent from other Asgard genomes? The authors mention a mosaic distribution of these enzymes but do not really discuss it.

We have now expanded the sentence describing the ester-linked lipids (lines 126-131). We also added the mosaic distribution of the genes across Asgard archaea in Fig. 1d.

Line 132: why is this intriguing?

The CRISPR/Cas systems has never been shown to have any particular global organization in the genomes, and we find it intriguing that they form this kind of 3-fold symmetry. However, as mentioned above with the restructuring in the current text, we opted to remove this description as we felt it was somewhat of a distraction to the flow and allowed us to focus more extensively on other elements in the manuscript. In the revised version, we also simplified the associated figure (originally figure 1a), by separating the distribution of CRISPR/Cas and the nontandem repeats in separate panels in Figure 2).

Line 137: did you try to reassemble by using your method?

The authors unfortunately did not submit the original sequencing reads to the public database.

Line 143: say more clearly that they are from bacteria and mention which bacteria.

A large part of the results presents an extensive analysis of integrated proviruses in the two circular genomes. This is very interesting and as I said, should be highlighted more in both the title and the abstract. Lines 250-256, please include a main figure and more detailed results to support your claims at lines 256-258).

We have now added mobile elements in the title and substantially streamlined the section regarding proviruses into two sections under CRISPR/Cas-guided discovery of Heimdallarchaeal mobile element (Fig. 3) and Diverse evolutionary origins of Heimdallarchaeal viruses (Fig. 4). The original lines 250-258, now expanded as a full section in line 237-267 supported by Fig. 3.

Line 305, the analysis of gene flux scaling is nice and new, and could certainly be further developed. The possibility that the genome sized of the other MAGs are affected by assembly problems is not evoked.

We have now expanded the analyses and narrative into a separate section (Genome-scalable gene flow across domains, lines 270-306) and a separate figure (Fig. 5). We think that providing functional analyses and a comparison with eukaryotic genomes can provide new insights.

We have now also added Figure S9 to specifically address the pitfall of MAGs with varying qualities. As shown in the Figure and explained in the text (299-301), incomplete and contaminated genomes completely obscured the constant archaeal fraction.

Line 330: interesting model, which could be merged with the last paragraph as a common discussion. However, as I said earlier, I do not think it is strongly supported by the data, or at least not as presented in the current text.

We intended for our hypothesized model be somewhat speculative based on the observation of extensive gene exchange across domain in the Asgard archaea, as well as the apparent fluidity of ESPs. The information contained in the new Fig. 5 and Fig. 6 hopefully provide further support for this idea. Our intension is for this illustration-assisted model to help general readers understand the context and to stimulate future discussions in the field. To better clarify the hypothetical nature of this, we now added the following in line 338-340 ‘We refer to this conceptual framework …’.

Please include a full etymology of the two candidatus at the end of the paper, so to protect yourself against a recent tendency by some people to change prokaryotic taxonomy (and names), creating a lot of confusion.

Thank you for this suggestion. We have now added an etymology section at the end of the paper.

Finally, be extra careful to provide all raw metagenomic data to the readership.

The genomes and metagenomic sequencing reads will be public under the Project No. PRJNA721962 upon the publication of the paper. It is now indicated in the Data availability section.

Decision Letter, first revision:

Dear Fabai, dear Victoria,

Thank you for submitting your revised manuscript "Unique mobile elements and scalable gene flow in archaea at the prokaryote-eukaryote boundary" (NMICROBIOL-21051233A). It has now been seen by the original referees and their comments are below. As you will see, the reviewers find that the paper has improved in revision, and therefore we'll be happy in principle to publish it in Nature Microbiology, pending some minor revisions to satisfy the referees' final requests and to comply with our editorial and formatting guidelines.

We are now performing detailed checks on your paper and will send you a checklist detailing all our editorial and formatting requirements once these are completed. These checks usually take around a week, but I will be away from the office next week, so will probably only be able to send you all these documents once I return, on October 25th. Please do not upload the final materials and make any revisions until you receive this additional information from us.

Thank you again for your interest in Nature Microbiology, and please do not hesitate to contact me if you have any questions.

Congratulations once again, and best regards,

{redacted}

****
Reviewer Comments:

Reviewer #1 (Remarks to the Author):

All of my comments have been addressed and I feel it's ready for publication.

****
Reviewer #2 (Remarks to the Author):

Wu et al. responded positively to the first round of revision, and this is an excellent manuscript that provides valuable new data and presents some conceptually interesting and provocative hypotheses about the prokaryote-to-eukaryote transition.

I have two minor points that the authors might wish to consider, both of which could be very quickly addressed.

1. I think "Andrew Rogers" in the acknowledgements should perhaps read "Andrew Roger", unless this refers to a less eminent phylogeneticist with a confusingly similar name.

2. In the revised manuscript, the section "Genome-scaleable gene flow across domains" presents a very interesting parallel between the chimeric genomes of Asgards and eukaryotes. I think the title of the section is perhaps difficult to understand, in the sense that this key idea (one of the main conceptual arguments of the paper) doesn't immediately jump out (since "genome-scaleable" is not very understandable, at least to me). I would suggest the authors might wish to change it, although this is of course their decision, not mine.

Something like "Parallels between genome chimerism in Asgard archaea and eukaryotes" or "The bacterial contribution to gene content scales with genome size in both Asgard archaea and eukaryotes" might get across the idea better.

****

Reviewer #3 (Remarks to the Author):

The authors have largely restructured the text, which now reads better and in a clearer way.

I am satisfied with it.

One important point is to provide not only ethymology but a full taxonomy for the two genomes (genus, family, order, phylum), so to establish them as the type strains for all Heimdall. Take a look at other recent papers that did that.

A few remaining typos:
line 47: in the resolution OF archaeal lineages...
line 87: collectively refer to them AS the Heimdall group...
line 92: formerly assigned under the Pacearchaeota, WHICH is...
line 97: and, GIVEN THE ABSENCE OF discernable terminal electron acceptors, MAY dissipate...
line 116: provide the full taxo too for H repetitus?
line 173: a feature often exploited by bacteriophages -> and archaeal viruses too?
lines 241-242: Bacteriodota or Bacteroidota?
line 247: While A majority of the viruses...
line 248: Caudivirales or Caudovirales?
line 385: I guess it is Andrew Roger not Rogers.

natureresearch

Decision Letter, final checks:

Dear Fabai, dear Victoria,

Thank you again for your patience as we've prepared the guidelines for final submission of your Nature Microbiology manuscript, "Unique mobile elements and scalable gene flow in archaea at the prokaryote-eukaryote boundary" (NMICROBIOL-21051233A). Please carefully follow the step-by-step instructions provided in the attached file, and add a response in each row of the table to indicate the changes that you have made. Ensuring that each point is addressed will help to ensure that your revised manuscript can be swiftly handed over to our production team.

We would like to start working on your revised paper, with all of the requested files and forms, as soon as possible (preferably within two weeks). Please get in contact with us if you anticipate delays.

When you upload your final materials, please include a point-by-point response to any remaining reviewer comments.

If you have not done so already, please alert us to any related manuscripts from your group that are under consideration or in press at other journals, or are being written up for submission to other journals (see: https://www.nature.com/nature-research/editorial-policies/plagiarism#policy-on-duplicate-publication for details).

In recognition of the time and expertise our reviewers provide to Nature Microbiology's editorial process, we would like to formally acknowledge their contribution to the external peer review of your manuscript entitled "Unique mobile elements and scalable gene flow in archaea at the prokaryote-eukaryote boundary". For those reviewers who give their assent, we will be publishing their names alongside the published article.

Nature Microbiology offers a Transparent Peer Review option for new original research manuscripts submitted after December 1st, 2019. As part of this initiative, we encourage our authors to support increased transparency into the peer review process by agreeing to have the reviewer comments, author rebuttal letters, and editorial decision letters published as a Supplementary item. When you submit your final files please clearly state in your cover letter whether or not you would like to participate in this initiative. Please note that failure to state your preference will result in delays in accepting your manuscript for publication.

Cover suggestions
As you prepare your final files we encourage you to consider whether you have any images or illustrations that may be appropriate for use on the cover of Nature Microbiology. Covers should be both aesthetically appealing and scientifically relevant, and should be supplied at the best quality available. Due to the prominence of these images, we do not generally select images featuring faces, children, text, graphs, schematic drawings, or collages on our covers. We accept TIFF, JPEG, PNG or PSD file formats (a layered PSD file would be ideal), and the image should be at least 300ppi resolution (preferably 600-1200 ppi), in CMYK colour mode. If your image is selected, we may also use it on the journal website as a banner image, and may need to make artistic alterations to fit our

journal style. Please submit your suggestions, clearly labeled, along with your final files. We'll be in touch if more information is needed.

Nature Microbiology has now transitioned to a unified Rights Collection system which will allow our Author Services team to quickly and easily collect the rights and permissions required to publish your work. Approximately 10 days after your paper is formally accepted, you will receive an email in providing you with a link to complete the grant of rights. If your paper is eligible for Open Access, our Author Services team will also be in touch regarding any additional information that may be required to arrange payment for your article.

Please note that you will not receive your proofs until the publishing agreement has been received through our system.

Please note that <i>Nature Microbiology</i> is a Transformative Journal (TJ). Authors may publish their research with us through the traditional subscription access route or make their paper immediately open access through payment of an article-processing charge (APC). Authors will not be required to make a final decision about access to their article until it has been accepted. Find out more about Transformative Journals

<B>Authors may need to take specific actions to achieve compliance with funder and institutional open access mandates. For submissions from January 2021, if your research is supported by a funder that requires immediate open access (e.g. according to Plan S principles) then you should select the gold OA route, and we will direct you to the compliant route where possible. For authors selecting the subscription publication route our standard licensing terms will need to be accepted, including our self-archiving policies. Those standard licensing terms will supersede any other terms that the author or any third party may assert apply to any version of the manuscript.

For information regarding our different publishing models please see our Transformative Journals page. If you have any questions about costs, Open Access requirements, or our legal forms, please contact ASJournals@springernature.com.

I think that's it. When you are ready, please use the following link for uploading all the required files and additional documents:

{redacted}

Thank you again for all your work on this, and I look forward to reading the final version. If you have any further questions, please feel free to contact me.

{redacted}

Final Decision Letter:

Dear Fabai, dear Victoria,

I am delighted to accept your Article "Unique mobile elements and scalable gene flow at the prokaryote-eukaryote boundary revealed by circularized Asgard archaea genomes" for publication in Nature Microbiology. Thank you for having chosen to submit your work to us and many congratulations to you and all your co-authors.

Before your manuscript is typeset, we will edit the text to ensure it is intelligible to our wide readership and conforms to house style. We look particularly carefully at the titles of all papers to ensure that they are relatively brief and understandable.

Once your manuscript is typeset and you have completed the appropriate grant of rights, you will receive a link to your electronic proof via email with a request to make any corrections within 48 hours. If, when you receive your proof, you cannot meet this deadline, please inform us at rjsproduction@springernature.com immediately. Once your paper has been scheduled for online publication, the Nature press office will be in touch to confirm the details.

Acceptance of your manuscript is conditional on all authors' agreement with our publication policies (see https://www.nature.com/nmicrobiol/editorial-policies). In particular your manuscript must not be published elsewhere and there must be no announcement of the work to any media outlet until the publication date (the day on which it is uploaded onto our website).

Please note that <i>Nature Microbiology</i> is a Transformative Journal (TJ). Authors may publish their research with us through the traditional subscription access route or make their paper immediately open access through payment of an article-processing charge (APC). Authors will not be required to make a final decision about access to their article until it has been accepted. Find out more about Transformative Journals

<B>Authors may need to take specific actions to achieve compliance with funder and institutional open access mandates. For submissions from January 2021, if your research is supported by a funder that requires immediate open access (e.g. according to Plan S principles) then you should select the gold OA route, and we will direct you to the compliant route where possible. For authors selecting the subscription publication route our standard licensing terms will need to be accepted, including our self-archiving policies. Those standard licensing terms will supersede any other terms that the author or any third party may assert apply to any version of the manuscript.

In approximately 10 business days you will receive an email with a link to choose the appropriate publishing options for your paper and our Author Services team will be in touch regarding any additional information that may be required.

natureresearch

You will not receive your proofs until the publishing agreement has been received through our system.

If you have any questions about our publishing options, costs, Open Access requirements, or our legal forms, please contact ASJournals@springernature.com

An online order form for reprints of your paper is available at https://www.nature.com/reprints/author-reprints.html. All co-authors, authors' institutions and authors' funding agencies can order reprints using the form appropriate to their geographical region.

We welcome the submission of potential cover material (including a short caption of around 40 words) related to your manuscript; suggestions should be sent to Nature Microbiology as electronic files (the image should be 300 dpi at 210 x 297 mm in either TIFF or JPEG format). Please note that such pictures should be selected more for their aesthetic appeal than for their scientific content, and that colour images work better than black and white or grayscale images. Please do not try to design a cover with the Nature Microbiology logo etc., and please do not submit composites of images related to your work. I am sure you will understand that we cannot make any promise as to whether any of your suggestions might be selected for the cover of the journal.

You can now use a single sign-on for all your accounts, view the status of all your manuscript submissions and reviews, access usage statistics for your published articles and download a record of your refereeing activity for the Nature journals.

To assist our authors in disseminating their research to the broader community, our SharedIt initiative provides you with a unique shareable link that will allow anyone (with or without a subscription) to read the published article. Recipients of the link with a subscription will also be able to download and print the PDF.

As soon as your article is published, you will receive an automated email with your shareable link.

Congratulations once again to you and your coauthors on putting together such a nice story, I look forward to seeing it published.
